# Regeneration programs buffer genetic defects in animal development

**DOI:** 10.1371/journal.pbio.3004011

**Published:** 2026-09-21

**Authors:** Kazunori Ando, Sushant Bangru, John Welsby, John D. Thompson, Kenneth D. Poss

**Affiliations:** 1 Morgridge Institute for Research, Madison, Wisconsin, United States of America; 2 Department of Cell and Regenerative Biology, University of Wisconsin--Madison, Madison, Wisconsin, United States of America; 3 Department of Cell Biology, Duke University School of Medicine, Durham, North Carolina, United States of America; Sloan Kettering Institute: Memorial Sloan Kettering Cancer Center, UNITED STATES OF AMERICA

## Abstract

Regeneration programs enable animals to restore damaged or lost tissues, and the range of stimuli for these programs is incompletely understood. Here, we used zebrafish, a vertebrate species with exceptional regenerative capacity, to identify chemically induced mutations that alter regeneration-associated gene activation. Transgenic zebrafish with a permissive promoter and EGFP cassette inserted in the vicinity of the pro-regenerative factor gene *fgf20a* were mutagenized, and larvae homozygous for ENU-induced mutations were assessed for disruptions in *fgf20a*-directed reporter gene expression following fin fold amputation. One line was identified with heritable, elevated *fgf20a*:*EGFP* presence in the absence of experimental injury, localized to regions of fin fold tissue undergoing degeneration. Whole-genome sequencing identified a mutation within exon 72 of the *fraser syndrome 1* (*fras1*) gene, mutated in patients with inherited skin disease. *fras1* mutant larvae spontaneously displayed broader signatures of regeneration, and zebrafish crispants for homologs of other genes mutated in human developmental diseases also displayed regeneration-associated gene expression in regions of dysmorphology. Tempering Fgf signaling by transgenic expression of a dominant-negative Fgf receptor in *fras1* mutants exacerbated the disease phenotype. Our findings provide evidence that regeneration programs are harnessed in response to developmental defects caused by genetic mutations, potentially buffering deleterious phenotypes.

## Abstract 2

Regeneration programs enable animals to restore damaged or lost tissues, but the stimuli that regulate these programs remain unclear. This study shows that mutations causing developmental defects activate regeneration responses that buffer the deleterious effects of congenital disease in zebrafish embryos.

## Introduction

One of the most striking biological disparities between vertebrate species is their varied capacity to regenerate damaged tissues. Whereas many adult mammalian tissues display fibrosis and incomplete repair in response to injury, organisms like salamanders and zebrafish can regenerate fins, limbs, heart, spinal cord, kidney, and other tissues/organs with remarkable fidelity [1,2]. Across these diverse tissue contexts, regeneration relies on establishing transcriptional signatures that trigger cell proliferation, direct tissue architecture, and restore tissue function. Broadly, these changes in gene expression that occur during regeneration, versus what normally is in place in the absence of injury, are referred to as “regeneration programs”. In recent years, it has become clear that regeneration programs are orchestrated by dedicated *cis*-regulatory elements [3,4]. First referred to as tissue regeneration enhancer elements (TREEs) and also called damage-responsive elements or regeneration-responsive elements, these sequences receive injury signals and selectively activate transcription in damaged tissue [3,5,6]. For example, the Fgf ligand gene *fgf20a* is induced within hours of fin amputation in zebrafish, where it is required to initiate formation of the regeneration blastema [7,8], and multiple TREEs appear to collectively direct its contextual expression [9]. Enhancers near *leptin*, *il11*, and *inhba* genes similarly direct target gene transcription after fish fin amputation [3,6,10]. Many other TREEs have been identified across diverse species, tissues, cells, and injury contexts [11–14].

A wide range of injury-associated cues can trigger regeneration programs. Reactive oxygen species, particularly hydrogen peroxide, form tissue-scale gradients within minutes of wounding and recruit leukocytes to initiate regenerative response [15]. Innate immune activity is similarly critical: macrophage-derived cytokines regulate blastema formation and patterning in both salamander limbs and zebrafish fins [16,17]. Oxygen levels provide another axis of control, as hypoxia can influence cardiomyocyte proliferation in zebrafish and neonatal mice [18,19]. Hormones are thought to gate regenerative competence; presence and activity of thyroid hormone levels suppress the capacity for cardiac regeneration, whereas disruption of thyroid hormone receptor signaling can enhance repair by altering metabolic and hypoxia pathways [20–22]. Innervation and mechanical strain are also early influences on regeneration [23–25]. Each of these triggering signals ultimately converges on regulatory controls like enhancers.

Fluctuations in signals with impact during regeneration also occur during normal development and tissue homeostasis. Tissue growth, turnover, and morphogenesis naturally involve episodes of hypoxia, mechanical strain, inflammation, and epithelial remodeling. The zebrafish heart, for example, undergoes a period of strain during the juvenile stage that initiates a program with several commonalities as injury-induced heart regeneration in adults [26]. During lung branching morphogenesis, rhythmic tissue expansion generates epithelial tension that activates YAP signaling to drive proliferation and epithelial remodeling—mechanisms like those redeployed during adult lung repair and regeneration [27]. Likewise, physiological hypoxia serves as a morphogenetic cue during early embryogenesis, where low oxygen tension enhances WNT pathway activity and primitive-streak gene expression through HIF-dependent regulation [28]. Thus, regeneration programs have the potential to act as surveillance systems, engaged at any point in life when tissues experience stress or compromised integrity—even during early development.

Here, we conducted a forward genetic screen using a zebrafish *fgf20a* enhancer trap line to find mutations that disrupt activation of regeneration-associated gene expression upon amputation injury in larval fins. Unexpectedly, we identified a line with recessive mutations in *fras1*, a gene previously linked to epithelial integrity and extracellular matrix (ECM) organization in species from zebrafish to humans, causing spontaneous activation of *fgf20a* regulatory sequences. To explore a hypothesis that enhancer-driven regenerative networks are deployed in response to intrinsic developmental defects, we analyzed additional regeneration-responsive gene expression in these mutants, performed crispant analysis of other genes associated with human developmental disorders, and experimentally limited Fgf signaling during *fras1* deficiency. Our results suggest a new and potentially important role for injury-induced gene expression programs as protective systems to buffer the effects of deleterious gene mutations.

## Results

### ENU mutagenesis screen for mutations that alter regeneration-associated *fgf20a* expression

Enhancer trap and reporter lines for injury-responsive genes enable real-time visualization of regeneration-associated gene expression and can be powerful tools for dissecting the molecular logic of regeneration (Fig 1A). To identify potential regulatory factors required for an essential factor for fin regeneration, *fgf20a*, we performed ENU mutagenesis in homozygous males of an *fgf20a* enhancer trap line (*HGn21A*, or *fgf20a*:*EGFP*; Fig 1B) [29]. In this line, a transposon-based *hsp70l*:*EGFP* cassette randomly inserted 1.5 kb upstream of the *fgf20a* gene locus. *fgf20a*:*EGFP* animals display strong, injury-induced EGFP expression after caudal fin amputation or larval fin fold amputation, mimicking endogenous *fgf20a* expression [30]. We treated 11 homozygous *fgf20a*:*EGFP* male fish (F_0_) with ENU, and, after confirming a high mutagenesis rate, we prepared 117 F_2_ families, all homozygous for *fgf20a*:*EGFP*, to breeding age by intercrossing 190 F_1_ fish (Fig 1C). Larval fin fold regeneration is robust in zebrafish, and both *fgf20a* mRNA and *fgf20a*:*EGFP* are induced within one day of amputation of the 3 day post fertilization (dpf) larval fin fold (Fig 1D and 1F). In total, we screened 9,868 total animals from 460 F3 families (all homozygous for *fgf20a*:*EGFP* and for ENU-induced mutations) for induction of *fgf20a*:*EGFP* after larval fin fold amputation.

**Fig 1:**
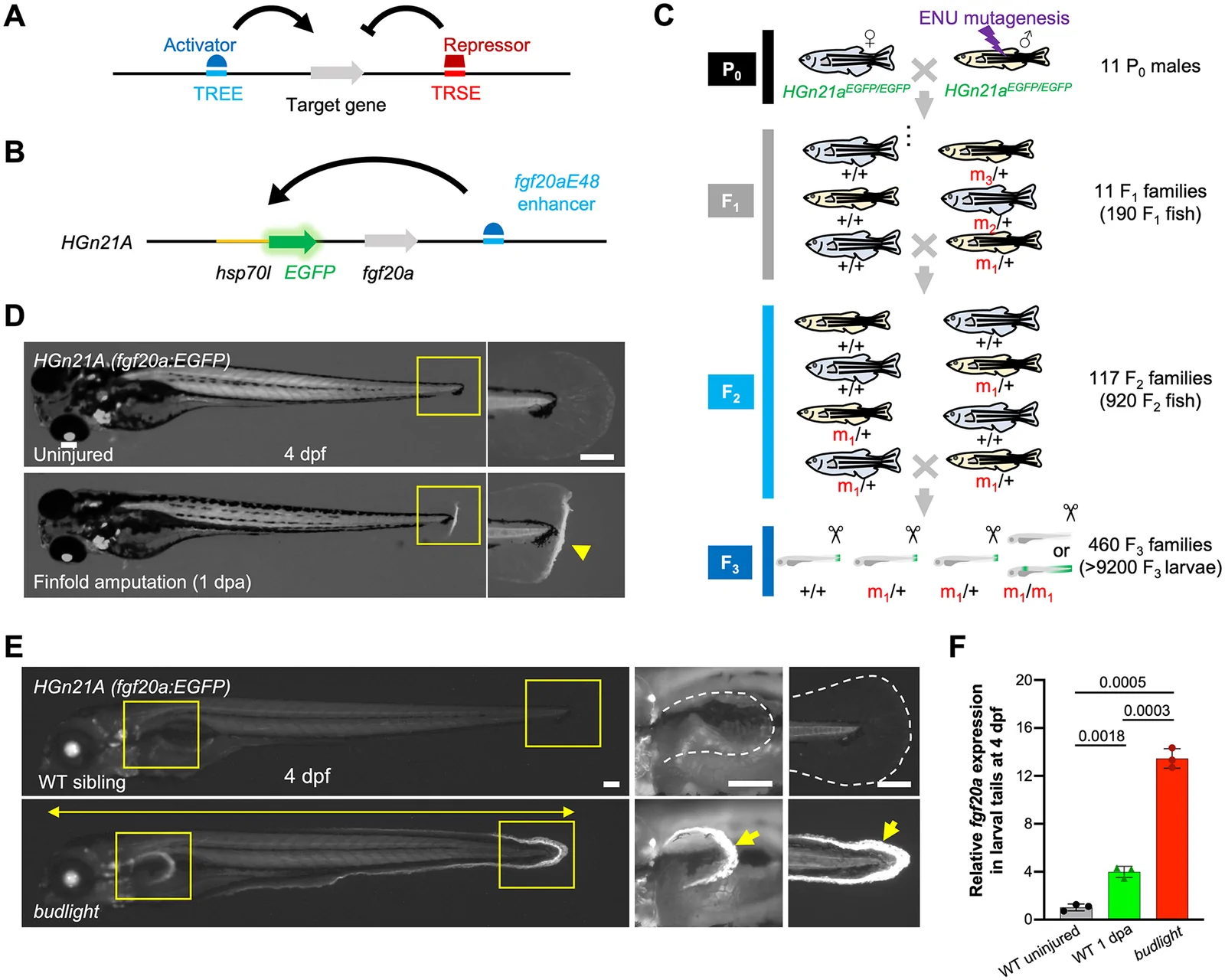
ENU mutagenesis identifies a mutation that increases *fgf20a*-directed expression at the degenerating fin fold. **(A)** Regeneration-responsive genes are regulated by tissue regeneration enhancer elements (TREEs) and tissue regeneration silencer elements (TRSEs). **(B)** Regeneration-responsive expression of the pro-regenerative factor, *fgf20a*, is regulated by a TREE, *fgf20aE48*, as present in the enhancer trap transgenic reporter line, *HGn21A*. **(C)** ENU mutagenesis screen flow. Homozygous male *HGn21A* (*fgf20a:EGFP*) fish were treated with ENU and mated with homozygous *fgf20a:EGFP* females to generate F_1_ families. F_2_ families were generated from intercrossing F_1_ fish, and screens of F_3_ larvae were performed to detect homozygous mutations that reduce or increase injury-induced fluorescence (m_1_, m_2_, m_3_,...). **(D)** (Top) Uninjured 4 dpf larva. (Bottom) 4 dpf larva after fin fold amputation at 3 dpf. EGFP expression is clearly induced at the injury site (yellow arrowhead in the right magnified view of tail region), mimicking *fgf20a* induction after the same injury. **(E)** The heritable mutant larvae (*budlight*) identified from screens spontaneously displayed a shortened body (yellow double-headed arrow) and elevated expression of *fgf20a*:*EGFP* in the degenerated fin fold (yellow arrows). **(F)** Quantification of relative expression levels of *fgf20a* in wild-type uninjured larvae, wild-type larvae at 1 day post-amputation (dpa), and *budlight* mutants at 4 dpf. Expression was significantly increased in wild-type larvae following injury and was highest in *budlight* mutants. Statistical significance was determined using a two-sample *t* test assuming unequal variances. *P* values are indicated above brackets. Data represent mean ± SEM. *n* = 3 pooled biological replicates per condition, with 20 larval tails pooled per replicate. Scale bars, 100 μm. The data underlying the graphs can be found at https://datadryad.org/search?q=10.5061%2Fdryad.jdfn2z3sm.

From these 460 families, 144 contained larvae with potential changes in *fgf20a:EGFP* reporter expression and/or defects in regeneration. In reassessments of these families, 11 displayed abnormal regeneration 1 day after fin fold amputation. Many families contained larvae displaying limited fin fold regeneration, but also with developmental defects like fin fold degeneration, cardiac edema, craniofacial malformation, or unusual trunk curvature (S1A and S1B Fig). Two of these families had members displaying subtle defects in fin regeneration at the adult stage -- apparent delays in initiating regeneration but recovery to normal regeneration (S1C Fig). Interestingly, while several families had increased *fgf20a:EGFP* in fin fold or trunk muscle, all of these families showed roughly normal *fgf20a:EGFP* expression at the amputation site. Only one of the families had members displaying a gross change in fin fold *fgf20a:EGFP* from clutchmates. These larvae displayed a jagged, degenerated fin fold in the absence of injury along with a conspicuous increase in *fgf20a:EGFP* at the caudal and ventral fin folds, and in pectoral fin buds, and thus we referred initially to this mutant as *budlight* (Fig 1E). qPCR analysis revealed that endogenous *fgf20a* levels are many-fold higher in *budlight* mutant tails than those of clutchmates (Fig 1F). To quantitatively assess fin fold degeneration and regeneration-associated signaling in *budlight* mutants, we measured fin fold area and EGFP reporter activity across larval development from 1–6 dpf (S2A and S2B Fig). Wild-type larvae displayed progressive expansion of fin fold tissue during normal development, whereas *budlight* mutants exhibited a pronounced failure of fin fold growth beginning around 2–3 dpf, resulting in reduced tissue area at later stages (S2A Fig). Concomitant with this degeneration phenotype, EGFP reporter intensity, normalized to tissue area, increased sharply in *budlight* mutants but remained low in wild-type larvae (S2B Fig). Notably, reporter activation coincided with the onset of overt tissue degeneration, suggesting that injury/regeneration-associated signaling programs are activated early during mutant fin fold degeneration. In summary, ENU mutagenesis revealed a heritable mutation that causes spontaneous elevation of the *fgf20a* regeneration program in uninjured fin primordia undergoing degeneration.

### A nonsense mutation in *fras1* causes ectopic *fgf20a*-directed gene expression

To identify the molecular basis of *budlight*, we performed whole-genome sequencing using genomic DNA from 5 dpf wild-type larvae homozygous for the *fgf20a:EGFP* insertion, as well as from *budlight* siblings displaying the degenerating fin fold phenotype (Fig 2A). Sequence reads were aligned to the zebrafish reference genome, and we applied filtering steps to remove background variants, sequencing artifacts, and low-quality calls. This filtering enriched for homozygous variants unique to the mutant pool, thereby prioritizing candidate lesions most likely to underlie the phenotype. SNP homozygosity analysis revealed a single point mutation in exon 72 of the *fraser extracellular matrix complex subunit 1* (*fras1*) gene, corresponding to the third-to-last exon. This variant represented a nonsense mutation (T to A), introducing a premature stop codon in place of tyrosine at amino acid position 3,708 (Fig 2B).

**Fig 2:**
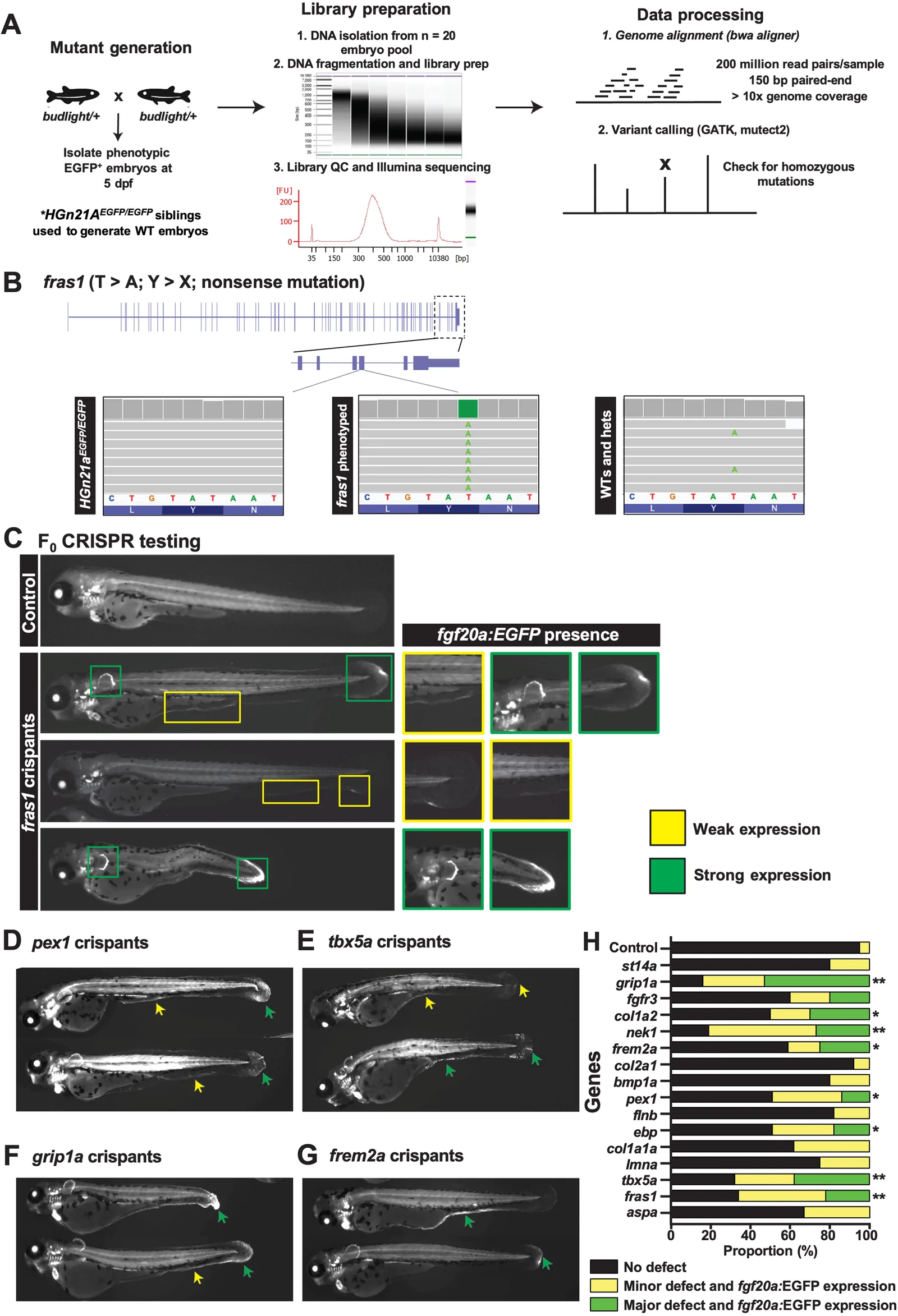
Identification of a nonsense mutation in *fras1*, and similar effects on *fgf20a* expression following genetic disruption of developmental disease-associated genes. **(A)** Workflow schematic for whole-genome sequencing-based identification of the causal mutation underlying the *budlight* phenotype. Homozygous mutant larvae displaying elevated *fgf20a*:*EGFP* expression were isolated from *budlight* heterozygous crosses at 5 dpf and pooled for genomic DNA extraction, library preparation, and Illumina sequencing. Sequence reads were aligned to the zebrafish reference genome followed by variant calling and identification of homozygous candidate mutations. **(B)** Whole-genome sequencing identified a nonsense mutation in *fras1* consisting of a T-to-A substitution in exon 72, converting a tyrosine codon into a premature stop codon. Representative sequencing alignments from mutant and sibling control samples are shown. **(C)** CRISPR-Cas9 F_0_ mutagenesis targeting *fras1* phenocopied the *budlight* mutant phenotype and induced ectopic *fgf20a*:*EGFP* expression. Representative examples of weak and strong reporter activation are shown in degenerating fin fold and craniofacial tissues. Control embryos displayed only canonical developmental *fgf20a*:*EGFP* expression domains. **(D–G)** Representative examples of phenotypes and ectopic *fgf20a*:*EGFP* activation observed following CRISPR-Cas9 F_0_ mutagenesis of zebrafish orthologues of human developmental disease-associated genes, including *pex1* (D), *tbx5a* (E), *grip1a* (F), and *frem2a* (G). Yellow arrows indicate regions with weak *fgf20a*:*EGFP* activation, and green arrows indicate regions with strong reporter induction. **(H)** Quantification of phenotypic classes observed in the CRISPR-Cas9 F_0_ screen. Larvae were categorized as displaying no detectable phenotype, minor defects with ectopic *fgf20a*:*EGFP* expression, or major defects with ectopic *fgf20a*:*EGFP* expression. Statistical significance was assessed using Fisher’s exact test with multiple testing correction. *p < 0.05, **p < 0.01. The data underlying the graphs can be found at https://datadryad.org/search?q=10.5061%2Fdryad.jdfn2z3sm.

*fras1* encodes a transmembrane protein with domains including von Willebrand factor–C and furin-like repeats, and it is crucial for epithelial–mesenchymal adhesion and basement membrane stability. In humans, mutations in FRAS1 cause Fraser syndrome, an autosomal recessive developmental disorder marked by epidermal blistering, syndactyly, and renal anomalies [31]. In zebrafish, *fras1* is expressed in epithelial tissues, particularly in developing fin folds, and is required for skin integrity and craniofacial morphogenesis [32]. Additionally, zebrafish mutants in *fras1*, as well as orthologues of FREM1/2 and other components of the Fraser syndrome complex, display classic fin blistering phenotypes that recapitulate aspects of the human and mouse diseases [33]. We observed blistering in *fras1* mutant fin folds at 1–2 dpf and not thereafter (S2C Fig), consistent with the defects of Fraser syndrome. We do not resolve whether posterior *fgf20a:EGFP* expression, and other responses described later, are responses to blister resolution and/or progressive degeneration of the affected tissue.

To independently test whether *fras1* disruption was responsible for the *fras1*^*pd416*^ degenerative phenotype and activation of the *fgf20a:EGFP* enhancer trap, we designed CRISPR/Cas9 guides targeting *fras1* coding sequences. Injection of gRNAs into one-cell stage embryos generated mosaic F_0_ animals that phenocopied the ENU mutant: at 3 dpf, injected embryos exhibited localized fin fold degeneration accompanied by ectopic *fgf20a* induction in the pectoral fin bud, ventral fin fold, and caudal fin fold (Fig 2C). These CRISPR experiments establish a causal link between *fras1* loss-of-function and the spontaneous activation of regeneration programs observed in *budlight* mutants, which we refer to hereafter as *fras1* mutants.

### Crispants in zebrafish orthologues of human congenital disorder genes show ectopic *fgf20a*-directed gene expression

Based on the association of *fras1* mutations with *fgf20a* induction, we postulated that, more broadly, intrinsic defects in tissue architecture that arise from genetic lesions in developmentally key genes can trigger regeneration programs. For example, intrinsic fragility or biomechanical stress as the result of mutations could activate regeneration programs that act as surveillance mechanisms. One prediction of this idea is that other genetic defects beyond *fras1* mutations should cause induction of regeneration-associated *fgf20a* expression.

To test if activation of regeneration-associated transcription is a general response to developmental defects, we performed a targeted loss-of-function (LOF) screen in the *fgf20a:EGFP* enhancer trap background. We selected 15 zebrafish orthologues of human disease genes that, when mutated in humans, cause congenital syndromes involving the skin, skeleton, or craniofacial structures—tissues related to those where *fgf20a* is induced by experimental injury (S3A Fig). These included genes linked to Fraser syndromes (*fras1*, *frem2*, *grip1*), osteogenesis imperfecta and related bone fragility (*bmp1a*, *col1a1*, *col1a2*, *col2a1*), skeletal dysplasia (*fgfr3*, *flnb*, *nek1*, *ebp*), limb and heart malformations (*tbx5a*), and cutaneous or connective tissue disorders (*st14*, *lmna*, *pex1*, *aspa*). We chose these genes based on documented human pathologies and evidence from zebrafish models: for instance, *fras1*/*pinfin* and *frem2*/*blasen* mutants present fin blistering [33], *bmp1a* mutants have ‘frilly fins’ owing to deficient collagen processing [34], *tbx5a*/*heartstrings* mutants exhibit pectoral fin and heart malformations [35], zebrafish *fgfr3* mutants show craniofacial malformations and delayed ossification similar to human skeletal dysplasia [36], and CRISPR-created *lmna* mutants present muscle and mobility defects analogous to muscular laminopathies [37].

Injection of CRISPR/Cas9 reagents targeting these genes resulted in larvae with varying degrees of morphological defects. In  every case where fin fold tissue integrity was visibly compromised, we observed ectopic activation of the *fgf20a:EGFP* enhancer trap in the affected regions (Figs 2D–2G, S3C and S3D). The induced EGFP expression domains extended beyond the normal developmental patterns of *fgf20a*, which typically include trunk muscle and craniofacial structures, and instead appeared in affected fin fold epithelia. Larvae that had been injected as embryos with Cas9 protein with or without non-targeting guide RNAs did not exhibit ectopic *fgf20a:EGFP* activation or overt tissue degeneration, indicating causal effects of specific gRNAs (S3B–S3D Fig). Statistical analyses indicated that approximately half of the tested genes produced a significant enrichment of larvae in the “defect plus ectopic *fgf20a* expression” category (Fig 2H). To complement the qualitative scoring of ectopic *fgf20a:EGFP* activation in F_0_ CRISPR embryos, we quantified fin fold tissue area and reporter activity at 4 dpf. Area measurements normalized to control larvae revealed that several perturbations identified by qualitative assessment as exhibiting strong enhancer activation and morphological defects also displayed significant tissue loss (S3E Fig). In parallel, integrated EGFP intensity normalized to fin fold area was elevated across most perturbations associated with ectopic reporter activation (S3F Fig), indicating enrichment of *fgf20a:EGFP* signal within the remaining fin fold tissue. Together, these quantitative analyses support the qualitative observation that developmental perturbations associated with tissue degeneration are accompanied by activation of *fgf20a* expression.

### Ectopic activation of broad regeneration responses in *fras1* mutant larvae

Another prediction of our hypothesis is that loss of *fras1* function should trigger regeneration-responsive transcriptional responses in addition to *fgf20a*. To test this prediction, we focused on two well-characterized TREEs, the first of which is *LEN*, which is required for regeneration-associated expression of the *leptin b* gene and directs expression of reporter transgenes in regenerating fin folds, adult fins, and adult hearts [3,9]. The second is *REN*, which is linked to the *runx1* locus and directs expression of reporter transgenes in injured and regenerating hearts; we found *REN* also directs regeneration-responsive expression in mesenchyme and notochord of *RENcfos:EGFP* larvae (Fig 3A and 3B) [3,38]. To test whether *fras1*-associated degeneration is sufficient to engage TREEs, we crossed *LENP2:EGFP* or *RENcfos:EGFP* reporter transgenes into the *fras1* background. We found that *fras1* mutants exhibited robust, ectopic activation of both reporters in affected fin fold tissue by 4 dpf, with patterns resembling their normal injury-induced expression domains (Fig 3C and 3D). This response was observed in all mutant larvae examined, whereas control siblings showed negligible detectable reporter expression in intact fin folds.

**Fig 3:**
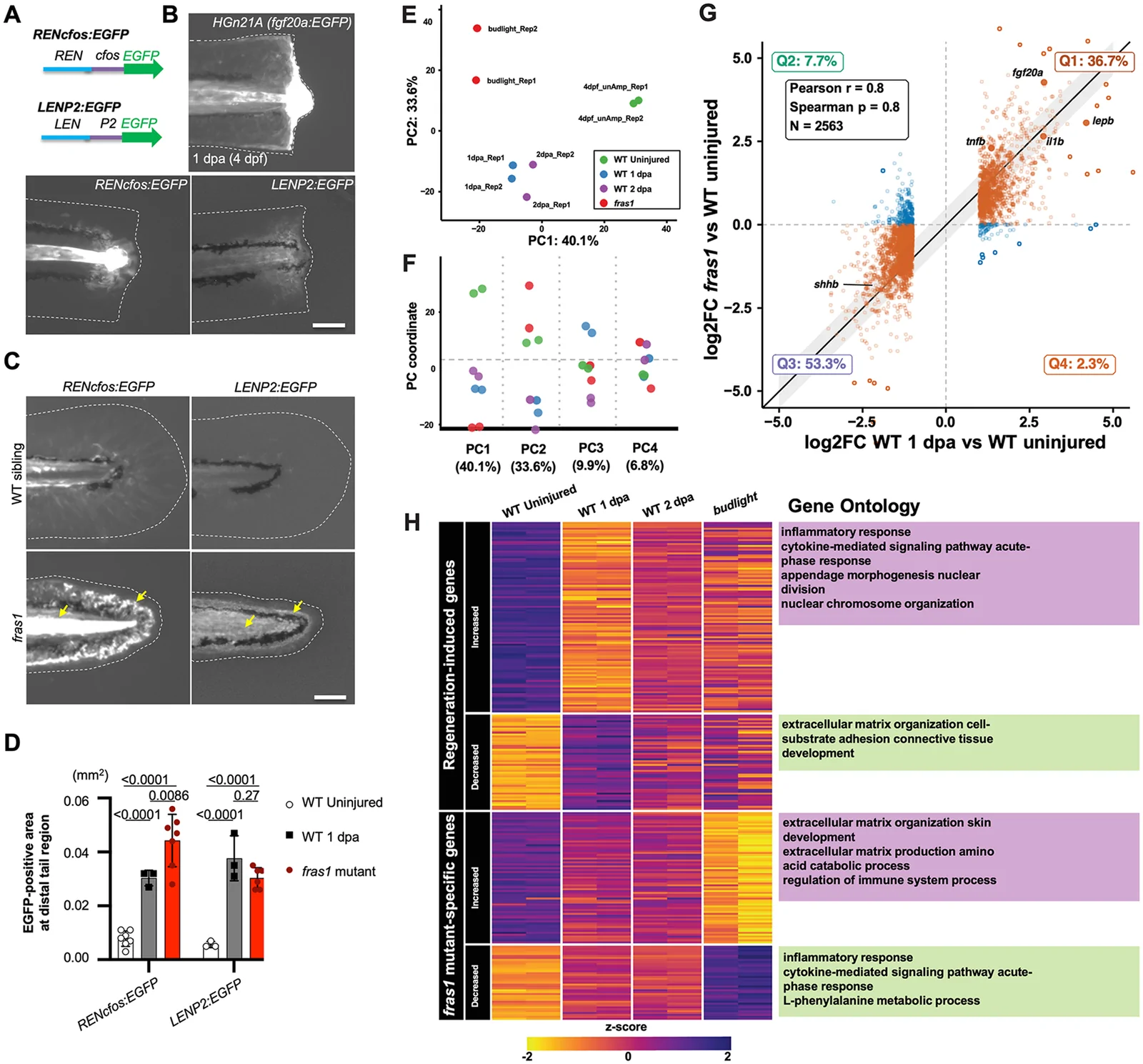
Evidence for a broad regeneration program activated in *fras1* mutants. **(A)** TREE reporter lines with *runx1* linked enhancer (*REN*) and *leptin b* linked enhancer (*LEN*) upstream of minimal promoter-EGFP cassettes. *cfos*, a 100 bp promoter of mouse *cfos*. *P2*, a 2 kb promoter of zebrafish *leptin b*. **(B)** Expression of reporters, *fgf20a:EGFP*, *RENcfos:EGFP* and *LENP2:EGFP* in regenerating larval fins at 4 dpf, after fin amputation at 3 dpf. *fgf20a:EGFP* has regeneration-responsive expression in epithelia, mesenchyme, notochord, and muscle, while *RENcfos:EGFP* and *LENP2:EGFP*, have regeneration-responsive expression in notochord and mesenchyme. Scale bars, 100 μm. **(C)** *fras1* mutations induce EGFP expression in notochord and mesenchyme (yellow arrows) of *RENcfos:EGFP* larvae and in mesenchyme (yellow arrows) of *LENP2:EGFP* larvae. Scale bars, 100 μm. **(D)** Measurement of EGFP-positive area at distal regions of 4 dpf tails in uninjured wild type (white bars), 1 dpa wild type (gray bars) and uninjured *fras1* mutants (red bars) with *RENcfos*:*EGFP* or *LENP2*:*EGFP* transgenes. Individual dots indicate biological replicates (*n* = 3–7). Data are presented as mean ± SEM. Statistical significance was assessed using two-way ANOVA. *P* values are shown above the corresponding comparisons. **(E)** Principal component analysis (PCA) of bulk RNA-seq datasets generated from wild-type (WT) uninjured larvae (4 dpf), wild-type regenerating tails at 1 and 2 dpa, and age-matched *fras1* mutant larvae. PC1 and PC2 account for 40.1% and 33.6% of total variance, respectively. **(F)** Sample coordinates across the first four principal components. Percentage variance explained for each principal component is indicated below the corresponding axis. **(G)** Correlation analysis comparing transcriptional changes during wild-type regeneration (1 dpa vs. wild-type uninjured) and *fras1* mutants (*fras1* vs. wild-type uninjured). Each point represents a single gene with |log_2_FC| > 1 during wild-type regeneration. Genes changing concordantly between regeneration and *fras1* mutants are shown in orange, while discordantly regulated genes are shown in blue. Representative regeneration-associated genes are labeled. Pearson and Spearman correlation coefficients are indicated. Shaded diagonal region denotes concordant fold-change directionality. **(H)** Heatmap showing transcriptional dynamics of representative regeneration-associated and *fras1* mutant-specific gene sets across wild-type uninjured, wild-type 1 dpa, wild-type 2 dpa, and *fras1* mutant conditions. Gene expression values are row-scaled z-scores. Adjacent panels summarize enriched Gene Ontology categories associated with each gene set. Displayed categories represent representative nonredundant functional groups selected after collapsing highly overlapping and related GO Biological Process terms. Complete gene-level differential-expression and GO-term assignments are provided in S3 Table. The data underlying the graphs can be found at https://datadryad.org/search?q=10.5061%2Fdryad.jdfn2z3sm.

To broadly test whether the fin fold degeneration observed in *fras1* mutants is associated with activation of regeneration programs, we performed bulk RNA-seq on isolated tail tissues from uninjured larvae (4 dpf), wild-type regenerating tails at 1 and 2 days post-amputation (dpa), and age-matched *fras1* mutant larvae at 4 dpf (S4A Fig). Principal component analysis (PCA) revealed that *fras1* transcriptomes clustered separately from uninjured wild-type controls and partially aligned with regenerating wild-type tissues along PC1, which accounted for 40.1% of total variance (Fig 3E and 3F). In contrast, PC2 (33.6% variance explained) distinguished regenerating wild-type samples from *fras1* mutants, suggesting that while the mutant engages regeneration-associated transcriptional programs, it also exhibits distinct transcriptional features. Consistent with this interpretation, gene ontology (GO) analysis of genes contributing to PC1 identified enrichment for regeneration-associated processes including ECM organization, connective tissue development, regeneration, and appendage morphogenesis, whereas PC2-associated genes were enriched for developmental and sensory perception pathways (S4B Fig).

To directly assess the relationship between regeneration and mutant transcriptional states, we compared gene expression changes between wild-type regeneration (1 dpa versus uninjured wild-type) and *fras1* mutants (*fras1* versus uninjured wild-type). Genes strongly altered during early regeneration exhibited a high degree of concordant regulation in *fras1* mutants (Pearson r = 0.8; Spearman ρ = 0.8; Fig 3G). Notably, 90% of regeneration-associated genes changed in the same direction in *fras1* mutants (Q1 + Q3 quadrants), including known regeneration-associated genes such as *lepb, fgf20a, il1b, tnfb,* and *shhb*. These findings suggest that *fras1* mutants spontaneously activate substantial components of the transcriptional program for regeneration in the absence of physical injury.

To further resolve shared and mutant-specific transcriptional responses, we classified genes based on whether they were similarly regulated during wild-type regeneration and in *fras1* mutants. Approximately 52.5% of transcriptionally altered genes were shared between regeneration and *fras1* conditions, while 25.9% represented *fras1*-specific changes and 21.6% were regeneration-associated genes with limited activation in the mutant background (S4C Fig). Heatmap analysis indicated that regeneration-associated genes were broadly upregulated in *fras1* mutants, particularly inflammatory and cytokine-associated pathways, whereas distinct *fras1-*specific programs included ECM organization, skin development, and metabolic pathways (Fig 3H). GO analysis further revealed enrichment of inflammatory response, cytokine-mediated signaling, and acute-phase response pathways among both regeneration-induced and *fras1*-downregulated gene sets, consistent with persistent activation of injury-associated signaling networks in the mutant state (Fig 3H). Together, these findings argue that loss of *fras1* gene function induces a transcriptional state that partially recapitulates injury-induced regeneration programs while also engaging mutant-specific developmental and ECM remodeling pathways.

### Genetic tempering of Fgf receptor signaling exacerbates *fras1* degenerative phenotypes

While evidence to this point indicates that genetic defects can cause regenerative responses in developing embryos, it was unclear if these responses have protective effects. Because *fgf20a* is ectopically induced in *fras1* mutants, we reasoned that Fgf signaling might be part of a compensatory response that helps tissues tolerate degeneration. We therefore tested both directions of pathway perturbation. To assess whether increasing Fgf signaling could ameliorate the phenotype, we attempted localized ligand overexpression. Ubiquitous Fgf ligand delivery is known to cause early patterning defects such as dorsalization [39], thus we used *itgb4* regulatory sequences—reported to drive expression in larval fin fold epithelia [40]—to confine *fgf20a* overexpression to the tissue of interest. Targeted *fgf20a* overexpression under *itgb4* control was not tolerated, producing embryonic lethality in F_0_ animals all of which were arrested at early stages and precluding assessment of improved responses (380 embryos assessed in two experiments). This is consistent with a narrow tolerance to elevated Fgf dosage even when spatially restricted.

In parallel, to test whether reducing Fgf signaling unmasks or worsens degeneration, we crossed *fras1* mutants to *hsp70l:dnfgfr1a-EGFP* animals, enabling heat-shock-inducible expression of a dominant-negative receptor (Figs 4A, 4B, and S5A) [41]. Temporally controlled Fgfr blockade by daily heat shock at larval stages shortened body length in both *fras1* mutants and control clutchmates, though more severely in transgenics than in controls. Additionally, *fras1* mutants displayed a higher frequency of posterior body distortion under Fgfr inhibition than their heat-shocked control siblings (Fig 4C–4E). Milder Fgfr blockade conditions (one heat shock every other day) did not affect body lengths; however, quantified fin fold areas were significantly decreased in *fras1* mutants but not control siblings (S5B–S5E Fig). Together, these results indicate that endogenous Fgf signaling is protective with respect to *fras1* mutant phenotypes—able to limit, but not prevent, tissue degeneration under the effects of genetic mutations.

**Fig 4:**
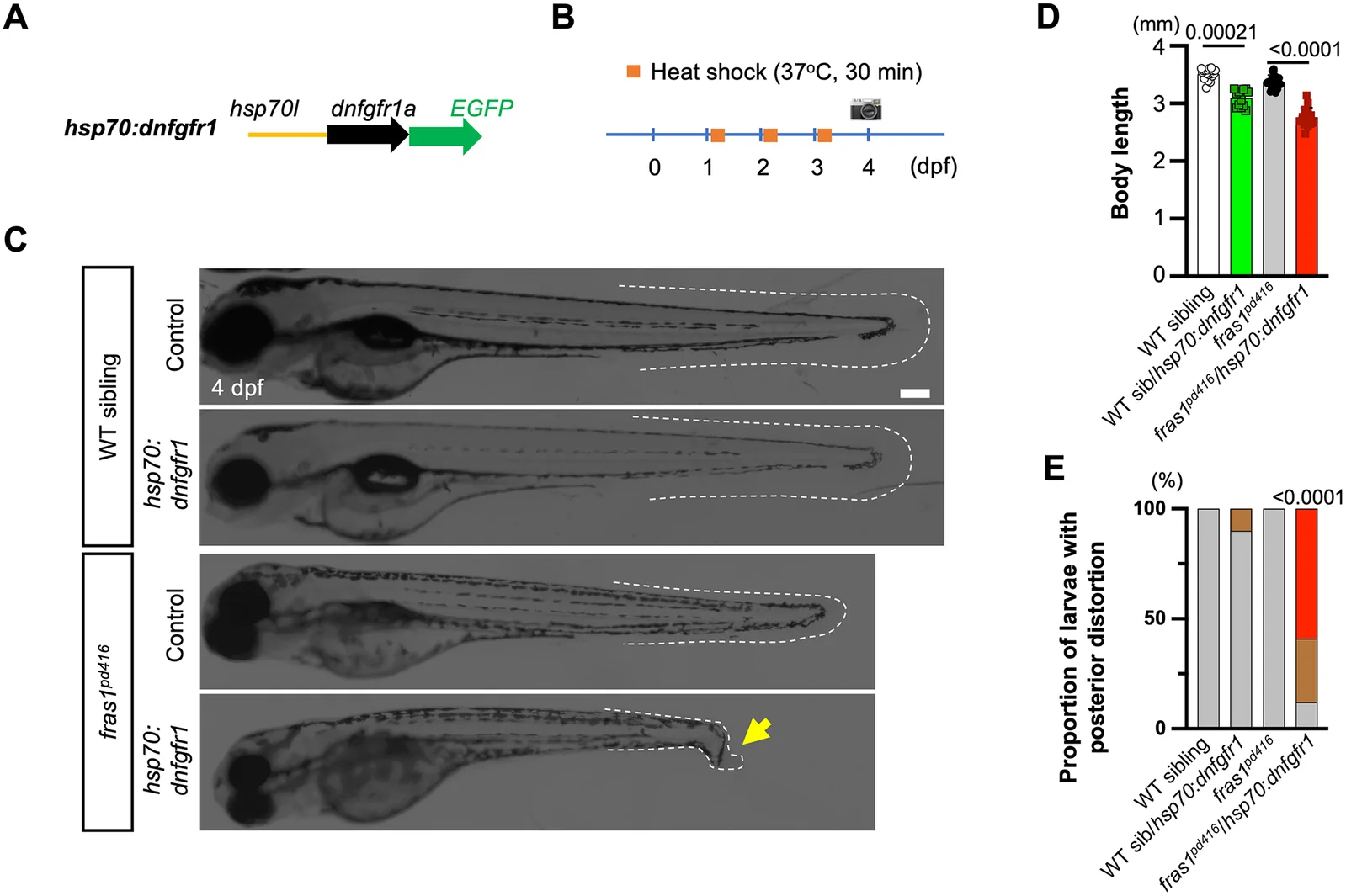
*fras1* phenotypes are exacerbated by Fgf receptor blockade. **(A)** Heat shock-inducible dominant negative Fgf receptor construct. **(B)** Heat shock is induced by incubating embryos in 37 °C eggwater for 30 min repeatedly at 1, 2, and 3 dpf, and larvae are assessed at 4 dpf. **(C)** Representative larvae of *hsp70*:*dnfgfr1* and *fras1* mutant lines and their negative controls at 4 dpf after three repeated heat shocks at 37 °C for 30 min. Scale bars, 100 μm. **(D, E)** Bar graphs of body lengths and proportions of larvae displaying a distorted posterior, from experiments in (C). Blocking Fgf signaling slightly shortens larval body lengths of wild-type and *fras1* mutant larvae, and *fras1* mutations sensitize the frequency at which posterior distortion is observed. Larvae were categorized as displaying no detectable phenotype, minor posterior distortion, or major posterior distortion. Data are presented as mean ± SEM or percentage. Statistical significance was assessed using two-way ANOVA or using Fisher’s exact test with multiple testing correction. *P* values are shown above the corresponding comparisons. The data underlying the graphs can be found at https://datadryad.org/search?q=10.5061%2Fdryad.jdfn2z3sm.

## Discussion

Our findings suggest a broader role for regeneration programs than previously appreciated. In addition to being deployed for homeostatic maintenance and in response to acute injury, regeneration-associated gene expression programs and signaling pathways, and, very importantly, enhancer gene regulatory elements, can be engaged by early developmental defects. This raises the possibility that regeneration modules function not only to restore tissue after loss, but also to buffer the inherent fragility of morphogenesis. Such a buffering role could help explain why regeneration programs are preserved in vertebrates like zebrafish: by responding to a wide range of stresses—including genetic mutations, mechanical perturbations, or epithelial instability—these programs may contribute to developmental robustness and organismal survival. In this sense, regeneration is not only a facultative repair response but a lifelong integrated component of tissue homeostasis and evolutionary fitness.

Several caveats temper this interpretation. The present work focuses on larval fin fold epithelia and a limited set of genes implicated in congenital disorders. We chose the fin fold as a sentinel tissue as the conceptual basis for this study emerged from an ENU screen focused on this tissue, and as the larval fin fold provides a sensitive and experimentally accessible tissue for detecting epithelial instability, morphogenetic disruption, and activation of regeneration-associated enhancers. It remains unclear whether similar regeneration-linked enhancer activation occurs broadly in internal organs or tissues more directly associated with the corresponding human congenital phenotypes, such as the heart, kidney, or endochondral skeleton. Our results also suggest that buffering may involve distinct genotypic and phenotypic classes. Mutations in structural and extracellular matrix genes (e.g., *fras1*, *frem2*) might compromise tissue integrity while ostensibly leaving the regenerative machinery intact, whereas some genes mutated in human congenital diseases are themselves components of the regenerative program: conditional inactivation of *tbx5a* in cardiomyocytes impairs zebrafish heart regeneration following ventricular resection [42]. Where the mutated gene is required for the response, the lesion compromises both the tissue and the capacity to compensate for it, and buffering would be predicted to fail. This framework generates a testable prediction: congenital defects of the first class should show greater variability in expressivity, whereas those of the second should be more uniformly penetrant.

Many but not all adult vertebrate tissues activate and require regeneration programs to replace day to day cell loss or wear and tear [2,43]. We have not tested how genetic lesions induced at the adult stage can be cloaked by regeneration programs, and we acknowledge that adult tissues may operate differently versus those undergoing initial development. Using conditional mutations or those inducing delayed-onset or progressive phenotypes could test this concept in mature tissues. Moreover, our assays identify enhancer activation and phenotypic buffering but do not yet reveal the upstream sensors of tissue instability or the molecular logic by which developmental stress is distinguished. These potential signals include mechanical stress involving ECM, inflammation associated with barrier failure, or factors released from dying cells. Future studies using single-cell transcriptomic and epigenomic approaches could clarify how regenerative circuits are rewired in genetic mutants, while cross-species comparisons may reveal whether the capacity to activate regeneration in response to developmental defects has been selectively maintained or diminished during evolution. Identifying these upstream signals and their integration with enhancer activity will be critical for testing if and how regeneration functions as a conserved developmental safeguard and whether this property can be leveraged therapeutically to ameliorate congenital disease.

The buffering mechanisms we describe are unlikely to be unique to highly regenerative species like zebrafish. Although adult mammals exhibit comparatively lower regenerative capacity, many human tissues retain regeneration-associated programs during development, juvenile growth, and specific pathological contexts. For example, skin and intestinal epithelia continuously regenerate through resident stem cell populations, skeletal muscle activates satellite-cell-mediated regeneration following injury and during early stages of muscular dystrophies, and the liver retains a substantial regenerative capacity throughout life. A mammalian genetic lesion can engage an injury program without experimental wounding: in keratin 5 (K5)-null mice, which undergo spontaneous epidermal cytolysis, the wound-healing-associated keratin K6 is induced in the suprabasal epidermis specifically in cytolyzed regions [44]. Mammalian fetal tissues display scar-free wound healing and broader regenerative responses that become progressively restricted during maturation. Together, these observations make it likely that regeneration-linked enhancers and signaling pathways also function in humans as partial buffering systems that mitigate developmental instability or tissue degeneration caused by congenital mutations, although more formal tests in mammalian contexts will be important. In this framework, the major difference between species is not presence versus absence of regenerative programs, but rather the competence, amplitudes, and range of tissue contexts in which these programs can be effectively deployed.

Many congenital mutations produce highly variable outcomes between individuals, even with identical genotypes. Such variability is thought to reflect compensatory mechanisms that mitigate developmental defects [45]. Regeneration programs, deployed through enhancer activation, may represent one such buffering system, limiting the impact of genetic lesions on tissue morphology. By connecting regeneration biology with developmental disease and variability, this work supports a model in which regeneration programs contribute to tissue robustness, and it highlights enhancer elements as a potential molecular interface between genetic lesions and organismal outcome.

## Materials and methods

### Zebrafish maintenance and husbandry

Adult zebrafish (Danio rerio) of the outbred Ekkwill (EK) strain, up to 18 months of age, were maintained under standard conditions. Fish were housed in a recirculating system on a 14 h light/10 h dark cycle and fed twice daily with a combination of live brine shrimp and commercial flake food. Water temperature was maintained at 26−28 °C, conductivity at 500–700 µS, and pH at 7.0–7.5. Wild-type or transgenic animals, including the *fgf20a* enhancer trap line (allele number *HGn21A*) and the *hsp70l:dnfgfr1a-EGFP* line (allele number *pd1)* were used as indicated [29,30,41]. The allele designation of the new *fras1* allele is *pd416*. All experiments were approved by and performed in accordance with institutional animal care and use protocols for zebrafish (M006843) at Duke University and the University of Wisconsin–Madison with animal assurance number D16-00239 (A3368-01).

### ENU mutagenesis in *fgf20a:EGFP* reporter fish

ENU mutagenesis was performed essentially as described [46,47]. Homozygous *fgf20a:EGFP* males (11 fish, ~9 months old) were treated weekly with 3.3 mM ENU for 1 h over 6 consecutive weeks in fish water containing 10 mM sodium phosphate buffer (pH 6.5). To assess mutation efficiency, treated males were test-crossed to females homozygous for recessive pigmentation mutations (*slc45a2*^*b4/b4*^ or *kita*^*b5/b5*^) which is easily scored by 3 dpf [48]. The ratio of pigmentation mutants was 4 in 1,323 (*slc45a2*^b4/^*) and 4 in 962 (*kita*^b5/^*). Mutagenized males were mated to *fgf20a*:*EGFP* homozygous females and generated 11 F_1_ families (a total of 190 F_1_ fish). Intercrossing of the F_1_ fish generated 117 F_2_ families (a total of 920 F_2_ fish). Intercrossing of pairs of F_2_ fish in each family provided 460 clutches of F_3_ embryos. Twenty larvae per family underwent fin fold amputation at 3 dpf, and regenerating fin folds were assessed at 4 dpf. Larvae with decreased or increased *fgf20a*:*EGFP* (or different EGFP patterns) at mendelian ratios were candidates for mutations affecting *fgf20a* regulation. Putative mutant families were out-crossed again to either EK wild-types or homozygous *fgf20a*:*EGFP* fish to generate F_4_ heterozygotes, which were in-crossed to generate F_5_ families. F_5_ families were used to confirm the phenotype.

### Anesthesia and larval fin fold amputation

For experimental manipulations, larvae were anesthetized in 0.02% tricaine methanesulfonate (MS-222, Sigma, buffered to pH 7.0 with Tris). To perform larval fin fold amputations, 3 dpf larvae were placed on an agar-coated Petri dish under a dissecting microscope. Using a sterile number 15 surgical scalpel, the fin fold was transected at the proximal edge of the pigment gap adjacent to the circulatory loop of the caudal vein. Larvae were transferred to fresh embryo medium and allowed to recover at 28 °C. Regeneration was assessed at 1 dpa by monitoring gross morphology and EGFP expression.

### Imaging

Whole-mount larval images were acquired using a Zeiss AxioZoom V16 stereo fluorescence microscope equipped with GFP filter sets. Larvae were anesthetized in 0.02% Tricaine and mounted laterally on a 1% low-melting agarose bed in embryo medium for imaging. Brightfield and fluorescence images were collected using identical exposure settings across groups for comparability. Images were processed using Zen (Zeiss) and Fiji (ImageJ) software.

### Whole-genome sequencing

For bulk whole-genome sequencing, *fras1* mutant carriers were crossed to produce clutches containing homozygous mutants and phenotypically wild-type siblings. At 5 dpf, pools of ~30 homozygous mutant larvae and ~30 siblings were collected. Genomic DNA was extracted using the DNeasy Blood and Tissue Kit (Qiagen, Cat. 69504) according to manufacturer’s protocol. Libraries were prepared using the NEBNext Ultra II FS DNA Library Prep Kit (NEB, Cat. E7805), quantified with Qubit dsDNA HS Assay (ThermoFisher), and fragment sizes were confirmed using a Bioanalyzer (Agilent). Sequencing was performed at BGI Genomics on a DNBseq platform with 150 bp paired-end reads, targeting at least 50 million read pairs per pool. Reads were trimmed with Trimmomatic, aligned to the Zebrafish GRCz11 reference genome using BWA-MEM, and variants were called with GATK HaplotypeCaller. Variants unique to mutant pools were identified, and filtering steps removed low-confidence calls, sequencing errors, and background polymorphisms from the EK strain. Homozygosity mapping identified a nonsense variant in *fras1*.

### Gene disruption by CRISPR/Cas9

CRISPR single-guide RNA (sgRNA) target sites were designed using the chopchop web tool (https://chopchop.cbu.uib.no/). Genomic DNA sequences retrieved from Ensembl GRCz10 or z11 (https://useast.ensembl.org/ Danio_rerio/Info/Index) were used for the target site searches. Target sequences were selected that had no predicted off-target sites in the reference genome and no mismatches at loci to be targeted based on the whole-genome sequencing data of the laboratory EK strain. Target-specific Alt-R crRNA and common Alt-R tracrRNA were synthesized by IDT, and each RNA was dissolved in duplex buffer (IDT) as 1 µg/µL stock solution. Stock solutions were stored at −80 °C. To prepare the crRNA:tracrRNA duplex, equal volumes of 1 µg/µL Alt-R crRNA and 1 µg/µL Alt-R tracrRNA stock solutions were mixed together and annealed by heating in a PCR machine: 95 °C, 5 min; followed by gradual cooling on the bench for a few minutes. The 1 µg/µL crRNA:tracrRNA duplex stock solution was mixed with equal volume of 1 µg/µL Cas9 protein (PNA BIO), incubated at 37 °C for 5 min, and kept on ice prior to injection. gRNA target sequences are listed in S1 Table.

### RNA isolation and quantitative PCR

Tail tissues from 20 larvae were pooled for each biological replicate and collected in Tri-Reagent (Sigma). RNA was isolated by a Direct-zol RNA Microprep Kit (Zymo). cDNA was synthesized from 100 ng of total RNA with a Maxima H Minus First Strand cDNA Synthesis Kit (Thermo). qPCR was performed on an Azure Cielo Real Time PCR system using LightCycler 480 SYBR Green I Master Mix (Roche) with primers listed in S2 Table. qPCR analyses of larval tail tissues of uninjured wild type, 1 dpa tail-amputated wild type, and *budlight* mutants were completed in technical triplicates at 4 dpf. All experiments normalized transcript expression levels to *actb2* as a housekeeping gene and then normalized the levels to uninjured wild type as a control. PRISM software was used to perform statistical analysis with paired *t* tests and generate graphs (GraphPad PRISM 11.0.1).

### Quantification of *RENcfos:EGFP* or *LENP2:EGFP* reporter activity

Wild-type (uninjured and 1 dpa) and *fras1* mutant larvae carrying the *RENcfos*:*EGFP* or *LENP2:EGFP* reporter transgene were imaged at 4 dpf using confocal microscopy. Tail tip regions were selected to include 500 μm anterior from the tip of notochord in Fiji/ImageJ. Thresholds were adjusted to detect only the EGFP-positive regions. Values were plotted as individual data points with mean ± SEM and assessed using two-way ANOVA.

### RNA-seq sample preparation and analysis

For bulk RNA sequencing, tail tissues were collected from WT uninjured larvae at 4 dpf, wild-type regenerating tails at 1 and 2 dpa, and age-matched *budlight* mutant larvae at 4 dpf. Tail amputations were performed at 2, and 3 dpf using a scalpel (Sterile Scalpel Blades #15, Feather). For each biological replicate, tail tissues from ~30–40 larvae were pooled and homogenized for total RNA extraction using Zymo RNA MicroPrep kit. RNA libraries were prepared using a nondirectional library preparation workflow and sequenced on an Illumina platform. Raw sequencing reads were processed to generate transcript-level abundance estimates, which were normalized as TPM (transcripts per million). TPM values were log2-transformed prior to downstream analysis. Principal component analysis (PCA) was performed using the top variable genes across samples. Gene ontology enrichment analysis was carried out using the clusterProfiler package with zebrafish annotations from org.Dr.eg.db. Correlation analyses comparing regeneration-associated and mutant-associated transcriptional changes were performed using Pearson and Spearman correlation coefficients calculated from log2 fold-change values relative to WT uninjured controls.

### Quantification of fin fold area and *fgf20a*:*EGFP* reporter activity

WT and *budlight* larvae carrying the *fgf20a*:*EGFP* reporter transgene were imaged daily from 1–6 dpf using Zeiss Axiozoom stereomicroscope. Fin fold tissue regions were manually segmented in Fiji/ImageJ to measure total fin fold area. Fin fold area was quantified in calibrated square microns (µm²) using microscope metadata imported through Bio-Formats in Fiji/ImageJ. EGFP reporter intensity was quantified within the segmented fin fold region using mean fluorescence intensity measurements and normalized to fin fold area to account for differences in tissue size between samples. Measurements were performed independently for each larva at each developmental stage, and values were plotted as individual data points with mean ± SEM.

For analysis of F_0_ CRISPR perturbation experiments, 4 dpf embryos carrying the *fgf20a:EGFP* enhancer trap were imaged on Zeiss Axiozoom stereomicroscope following injection of Cas9 RNP complexes targeting candidate genes or control reagents. In cases where multiple larvae were present within a single image, individual fin fold ROIs were manually segmented separately for each embryo using Fiji/ImageJ. Fin fold area, mean EGFP intensity, and integrated EGFP intensity were measured from the segmented ROIs using raw fluorescence values. Fin fold area measurements were normalized to the mean fin fold area of control-injected embryos. Integrated EGFP intensity was additionally normalized to fin fold area, and then normalized again to the control average, and plotted as individual larval data points with mean ± SEM. Statistical comparisons between conditions were performed using Welch’s t-tests with Benjamini–Hochberg false discovery rate (FDR) correction.

### *hsp70:dnfgfr1* experiments

Embryos from crosses of *hsp70*:*dnfgfr1*^+/-^;*fras1*^*pd416*/+^ and *fras1*^*pd416*/+^ fish were all heat-shocked by transfer into pre-warmed egg water for 30 min at 37 °C, followed by transfer into egg water at room temperature. Heat shock was performed every day (1, 2, and 3 dpf) or every other day (2, 4, and 6 dpf) and the phenotype changes were assessed at 4 dpf or 2, 4, 6, and 8 dpf. Anesthetized larvae were imaged using a Zeiss AxioZoom microscope. All raw images were processed using either Zen (Zeiss) or Fiji software. Larval body lengths were measured using Fiji software as a longest straight line spanning the body projection. Proportions of larvae with posterior distortion after Fgfr blockade were calculated based on the numbers of larvae with or without a mild/severe phenotype at the posterior region and analyzed using Fisher’s exact test.

## Supporting information

S1 Fig Phenotypes of F_3_ larvae and adults. **(A)** Ten F_3_ families containing larvae with limited regeneration phenotypes (yellow arrowheads) associated with EGFP expression, in both 1st and 2nd repeated screenings. F_3_ families 1–5 displayed reduced size of regenerating tissues, and F_3_ families 5–9 displayed EGFP at the edge of fin folds. *budlight* mutants exhibited a strong regeneration defect but also degenerated fin folds in the absence of injury. Scale bar, 100 μm. **(B)** Ratios of larvae showing phenotypes (brown) from 25–35 screened F_3_ larvae for each family shown in (A). Most F_3_ families displayed ratios consistent with recessive transmission. **(C)** Two families from (A) contained adult fish displayed abnormal caudal fin regeneration (yellow arrowheads) at 2 dpa (left panels), although fin regeneration was grossly normal at 7 dpa (right panels). White arrowheads indicate amputation planes. Scale bar, 1 mm. The data underlying the graphs can be found at https://datadryad.org/search?q=10.5061%2Fdryad.jdfn2z3sm.
(TIF)

S2 Fig Quantification of fin fold degeneration and *fgf20a*:*EGFP* reporter activation in *budlight* mutants. **(A)** Quantification of fin fold area in wild-type and *budlight* larvae from 1–6 dpf. Fin fold area was measured from maximum intensity projections generated from confocal z-stacks. Wild-type larvae exhibited progressive fin fold expansion during development, whereas *budlight* mutants plateaued by 2 dpf. Area values on y-axis represent fin fold area in µm². Each point represents an individual larva; error bars indicate mean ± SEM. **(B)** Quantification of *fgf20a*:*EGFP* reporter intensity normalized to fin fold area in wild-type and *budlight* larvae from 1–6 dpf. EGFP fluorescence intensity was measured within the segmented fin fold region and normalized to tissue area. *budlight* mutants exhibited increased reporter activity relative to wild-type controls beginning at ~2–3 dpf. Each point represents an individual larva; error bars indicate mean ± SEM. **(C)** *budlight* mutants displayed characteristic temporary blistering (white arrows) within the fin fold during 1–2 dpf. Blisters were no longer visible at 3 dpf when the fin folds were severely degenerated. Scale bar, 100 μm. The data underlying the graphs can be found at https://datadryad.org/search?q=10.5061%2Fdryad.jdfn2z3sm.
(TIF)

S3 Fig CRISPR-Cas9 F0 screen of developmental disease-associated genes. **(A)** Table of human disease-associated genes included in the CRISPR-Cas9 F_0_ mutagenesis screen and their corresponding human disorders. **(B)** Representative images of *fgf20a*:*EGFP* larvae uninjected as embryos, injected with Cas9 protein alone, or injected with Cas9 protein together with a nontargeting guide RNA. Neither Cas9-only or nontargeting guide injections induced ectopic *fgf20a:EGFP* expression or overt tissue degeneration. **(C, D)** Additional representative examples of ectopic *fgf20a*:*EGFP* activation following CRISPR-Cas9 mutagenesis of *flnb* (C) and *aspa* (D). Yellow arrows indicate regions displaying ectopic reporter activation within degenerating fin fold tissues. **(E)** Fin fold area measurements normalized to the mean control fin fold area for each condition. **(F)** Integrated EGFP intensity normalized to fin fold area, and then to the average control value, for each condition. Individual points represent single larvae; bars indicate mean ± SEM. Statistical significance was determined relative to controls using Welch’s t-tests with Benjamini–Hochberg FDR correction (*FDR < 0.05, **FDR < 0.01, ***FDR < 0.001, ****FDR < 0.0001). The data underlying the graphs can be found at https://datadryad.org/search?q=10.5061%2Fdryad.jdfn2z3sm.
(TIF)

S4 Fig Transcriptome analysis workflow and supplementary analyses of regeneration-associated gene programs. **(A)** Schematic overview of RNA-seq experimental design. Tail amputations were performed at 3 dpf, followed by tissue collection from wild-type (WT) uninjured larvae (4 dpf), WT regenerating tails at 1 and 2 dpa, and age-matched *fras1* mutant larvae. Tail tissues from 30–40 larvae were pooled for RNA isolation and nondirectional library preparation. **(B)** Gene ontology enrichment analysis of genes contributing to PC1 and PC2 from principal component analysis. PC1-associated genes were enriched for ECM organization, connective tissue development, regeneration, and skeletal development pathways, whereas PC2-associated genes were enriched for developmental and sensory perception-related processes. Dot size indicates gene count and color denotes adjusted *p*-value. **(C)** Correlation analysis comparing transcriptional changes during WT regeneration and *fras1* mutants, including genes altered in either or both condition and excluding those without changes in either. Genes were classified as shared, WT regeneration-specific, or *fras1*-specific based on fold-change directionality and magnitude thresholds (|log_2_FC| ≥ 1, FDR corrected *p*-value < 0.05). Pearson and Spearman correlation coefficients and category frequencies are indicated within the plot. https://datadryad.org/search?q=10.5061%2Fdryad.jdfn2z3sm.
(TIF)

S5 Fig *fras1* skin degeneration phenotypes are exacerbated by mild Fgf receptor blockade. **(A)** Representative larvae of *hsp70*:*dnfgfr1* and *fras1* mutant lines at 3 dpf before heat shock and at 4 dpf after heat shock at 37 °C for 30 min at 3 dpf. Fin fold degeneration in *fras1* mutants does not activate *hsp70* promoter without heat shock (yellow arrow). **(B)** Heat shocks were performed every other day at 2, 4, and 6 dpf, with monitoring at 2, 4, 6, and 8 dpf. **(C)** Representative *hsp70*:*dnfgfr1* and *fras1* mutant larvae and their negative controls at 2, 4, 6 and 8 dpf. *fras1* mutant fin fold sizes appeared slightly reduced in the milder heat shock condition (yellow arrows). **(D, E)** Measurement of body lengths and fin fold areas of larvae in these experiments. White and green bars represent wild-type siblings with/without the *hsp70*:*dnfgfr1* transgene, showing no significant difference in body lengths or fin fold areas from each other. Gray and red bars represent *fras1* mutants with/without the *hsp70*:*dnfgfr1* transgene, with measurable reductions in fin fold area caused by Fgfr blockade. Individual points indicate biological replicates (*n* = 6). Data are presented as mean ± SEM. Statistical significance was assessed using two-sample *t*-tests assuming unequal variances, or Fisher’s exact test with correction for multiple comparisons. *P* values are shown above the corresponding comparisons. Scale bars, 100 μm. https://datadryad.org/search?q=10.5061%2Fdryad.jdfn2z3sm.
(TIF)

S1 Table Guide RNA targeting sequences (length, 20 nt) used for the F_0_ screen of zebrafish orthologues of human congenital disease-associated genes. For all genes, a combination of 2–3 guide RNAs in equimolar quantities was used.
(CSV)

S2 Table Primers used for qPCR analysis of relative *fgf20a* expression in *budlight* mutants and wild-type siblings. (CSV)

S3 Table Differentially expressed genes and associated Gene Ontology (GO) categories identified from transcriptomic analysis of wild-type regeneration and *budlight* mutants. The table includes gene identifiers, gene symbols, log2 fold changes, adjusted *P* values, and assignment to enriched GO Biological Process terms and representative nonredundant functional categories displayed in Fig 3H.
(XLSX)

## Abbreviations

dpa: days post-amputation
dpf: day post fertilization
ECM: extracellular matrix
FDR: false discovery rate
GO: Gene Ontology
K5: keratin 5
LEN: linked enhancer
LOF: loss-of-function
PCA: Principal component analysis
REN: runx1 linked enhancer
TREEs: tissue regeneration enhancer elements
TRSEs: tissue regeneration silencer elements
WT: wild-type.

## References

[1] Iismaa SE, Kaidonis X, Nicks AM, Bogush N, Kikuchi K, Naqvi N, et al. Comparative regenerative mechanisms across different mammalian tissues. NPJ Regen Med. 2018;3:6. doi: 10.1038/s41536-018-0044-5 29507774 PMC5824955

[2] Poss KD, Tanaka EM. Hallmarks of regeneration. Cell Stem Cell. 2024;31(9):1244–61. doi: 10.1016/j.stem.2024.07.007 39163854 PMC11410156

[3] Kang J, Hu J, Karra R, Dickson AL, Tornini VA, Nachtrab G, et al. Modulation of tissue repair by regeneration enhancer elements. Nature. 2016;532(7598):201–6. doi: 10.1038/nature17644 27049946 PMC4844022

[4] Goldman JA, Poss KD. Gene regulatory programmes of tissue regeneration. Nat Rev Genet. 2020;21(9):511–25. doi: 10.1038/s41576-020-0239-7 32504079 PMC7448550

[5] Harris RE, Setiawan L, Saul J, Hariharan IK. Localized epigenetic silencing of a damage-activated WNT enhancer limits regeneration in mature Drosophila imaginal discs. Elife. 2016;5:e11588. doi: 10.7554/eLife.11588 26840050 PMC4786413

[6] Wang W, Hu C-K, Zeng A, Alegre D, Hu D, Gotting K, et al. Changes in regeneration-responsive enhancers shape regenerative capacities in vertebrates. Science. 2020;369(6508):eaaz3090. doi: 10.1126/science.aaz3090 32883834 PMC9479427

[7] Whitehead GG, Makino S, Lien C-L, Keating MT. fgf20 is essential for initiating zebrafish fin regeneration. Science. 2005;310(5756):1957–60. doi: 10.1126/science.1117637 16373575

[8] Shibata E, Yokota Y, Horita N, Kudo A, Abe G, Kawakami K, et al. Fgf signalling controls diverse aspects of fin regeneration. Development. 2016;143(16):2920–9. doi: 10.1242/dev.140699 27402707

[9] Thompson JD, Ou J, Lee N, Shin K, Cigliola V, Song L, et al. Identification and requirements of enhancers that direct gene expression during zebrafish fin regeneration. Development. 2020;147(14):dev191262. doi: 10.1242/dev.191262 32665240 PMC7406312

[10] Chen Y, Hou Y, Zeng Q, Wang I, Shang M, Shin K, et al. Common and specific gene regulatory programs in zebrafish caudal fin regeneration at single-cell resolution. Genome Res. 2025;35(1):202–18. doi: 10.1101/gr.279372.124 39809530 PMC11789645

[11] Cigliola V, Shoffner A, Lee N, Ou J, Gonzalez TJ, Hoque J, et al. Spinal cord repair is modulated by the neurogenic factor Hb-egf under direction of a regeneration-associated enhancer. Nat Commun. 2023;14(1):4857. doi: 10.1038/s41467-023-40486-5 37567873 PMC10421883

[12] Jimenez E, Slevin CC, Song W, Chen Z, Frederickson SC, Gildea D, et al. A regulatory network of Sox and Six transcription factors initiate a cell fate transformation during hearing regeneration in adult zebrafish. Cell Genom. 2022;2(9):100170. doi: 10.1016/j.xgen.2022.100170 36212030 PMC9540346

[13] Lin W, Jia X, Shi X, He Q, Zhang P, Zhang X, et al. Reactivation of mammalian regeneration by turning on an evolutionarily disabled genetic switch. Science. 2025;388(6754):eadp0176. doi: 10.1126/science.adp0176 40570123

[14] Shi T, Kim Y, Llamas J, Wang X, Fabian P, Lozito TP, et al. Long-range Atoh1 enhancers maintain competency for hair cell regeneration in the inner ear. Proc Natl Acad Sci U S A. 2024;121(51):e2418098121. doi: 10.1073/pnas.2418098121 39671177 PMC11665905

[15] Niethammer P, Grabher C, Look AT, Mitchison TJ. A tissue-scale gradient of hydrogen peroxide mediates rapid wound detection in zebrafish. Nature. 2009;459(7249):996–9. doi: 10.1038/nature08119 19494811 PMC2803098

[16] Godwin JW, Pinto AR, Rosenthal NA. Macrophages are required for adult salamander limb regeneration. Proc Natl Acad Sci U S A. 2013;110(23):9415–20. doi: 10.1073/pnas.1300290110 23690624 PMC3677454

[17] Petrie TA, Strand NS, Yang C-T, Rabinowitz JS, Moon RT. Macrophages modulate adult zebrafish tail fin regeneration. Development. 2014;141(13):2581–91. doi: 10.1242/dev.098459 24961798 PMC4067955

[18] Jopling C, Suñé G, Faucherre A, Fabregat C, Belmonte JCI. Hypoxia induces myocardial regeneration in zebrafish. Circulation. 2012;126(25):3017–27. doi: 10.1161/circulationaha.112.107888 23151342

[19] Puente BN, Kimura W, Muralidhar SA, Moon J, Amatruda JF, Phelps KL, et al. The oxygen-rich postnatal environment induces cardiomyocyte cell-cycle arrest through DNA damage response. Cell. 2014;157(3):565–79. doi: 10.1016/j.cell.2014.03.032 24766806 PMC4104514

[20] Hirose K, Payumo AY, Cutie S, Hoang A, Zhang H, Guyot R, et al. Evidence for hormonal control of heart regenerative capacity during endothermy acquisition. Science. 2019;364(6436):184–8. doi: 10.1126/science.aar2038 30846611 PMC6541389

[21] Cheung MY, Jiang C, Hassan IU, Wang H, Guo D, Dio DW, et al. Knockout of thyroid hormone receptor alpha a (thraa) enhances cardiac regeneration in zebrafish through metabolic and hypoxic regulation. Cell Commun Signal. 2025;23(1):340. doi: 10.1186/s12964-025-02350-5 40671086 PMC12265366

[22] Ross I, Omengan DB, Huang GN, Payumo AY. Thyroid hormone-dependent regulation of metabolism and heart regeneration. J Endocrinol. 2022;252(3):R71–82. doi: 10.1530/JOE-21-0335 34935637 PMC8776588

[23] Todd TJ. Physiological discoveries. Br Foreign Med Rev. 1837;4(7):281–2. 30161890 PMC5595466

[24] Kumar A, Godwin JW, Gates PB, Garza-Garcia AA, Brockes JP. Molecular basis for the nerve dependence of limb regeneration in an adult vertebrate. Science. 2007;318(5851):772–7. doi: 10.1126/science.1147710 17975060 PMC2696928

[25] Engler AJ, Sen S, Sweeney HL, Discher DE. Matrix elasticity directs stem cell lineage specification. Cell. 2006;126(4):677–89. doi: 10.1016/j.cell.2006.06.044 16923388

[26] Gupta V, Gemberling M, Karra R, Rosenfeld GE, Evans T, Poss KD. An injury-responsive gata4 program shapes the zebrafish cardiac ventricle. Curr Biol. 2013;23(13):1221–7. doi: 10.1016/j.cub.2013.05.028 23791730 PMC3759223

[27] Lin C, Yao E, Zhang K, Jiang X, Croll S, Thompson-Peer K, et al. YAP is essential for mechanical force production and epithelial cell proliferation during lung branching morphogenesis. Elife. 2017;6:e21130. doi: 10.7554/eLife.21130 28323616 PMC5360446

[28] López-Anguita N, Gassaloglu SI, Stötzel M, Bolondi A, Conkar D, Typou M, et al. Hypoxia induces an early primitive streak signature, enhancing spontaneous elongation and lineage representation in gastruloids. Development. 2022;149(20):dev200679. doi: 10.1242/dev.200679 36102628 PMC9578691

[29] Nagayoshi S, Hayashi E, Abe G, Osato N, Asakawa K, Urasaki A, et al. Insertional mutagenesis by the Tol2 transposon-mediated enhancer trap approach generated mutations in two developmental genes: tcf7 and synembryn-like. Development. 2008;135(1):159–69. doi: 10.1242/dev.009050 18065431

[30] Shibata E, Ando K, Murase E, Kawakami A. Heterogeneous fates and dynamic rearrangement of regenerative epidermis-derived cells during zebrafish fin regeneration. Development. 2018;145(8):dev162016. doi: 10.1242/dev.162016 29615465

[31] McGregor L, Makela V, Darling SM, Vrontou S, Chalepakis G, Roberts C, et al. Fraser syndrome and mouse blebbed phenotype caused by mutations in FRAS1/Fras1 encoding a putative extracellular matrix protein. Nat Genet. 2003;34(2):203–8. doi: 10.1038/ng1142 12766769

[32] Talbot JC, Walker MB, Carney TJ, Huycke TR, Yan Y-L, BreMiller RA, et al. fras1 shapes endodermal pouch 1 and stabilizes zebrafish pharyngeal skeletal development. Development. 2012;139(15):2804–13. doi: 10.1242/dev.074906 22782724 PMC3392706

[33] Carney TJ, Feitosa NM, Sonntag C, Slanchev K, Kluger J, Kiyozumi D, et al. Genetic analysis of fin development in zebrafish identifies furin and hemicentin1 as potential novel fraser syndrome disease genes. PLoS Genet. 2010;6(4):e1000907. doi: 10.1371/journal.pgen.1000907 20419147 PMC2855323

[34] Asharani PV, Keupp K, Semler O, Wang W, Li Y, Thiele H, et al. Attenuated BMP1 function compromises osteogenesis, leading to bone fragility in humans and zebrafish. Am J Hum Genet. 2012;90(4):661–74. doi: 10.1016/j.ajhg.2012.02.026 22482805 PMC3322236

[35] Garrity DM, Childs S, Fishman MC. The heartstrings mutation in zebrafish causes heart/fin Tbx5 deficiency syndrome. Development. 2002;129(19):4635–45. doi: 10.1242/dev.129.19.4635 12223419

[36] Sun X, Zhang R, Chen H, Du X, Chen S, Huang J, et al. Fgfr3 mutation disrupts chondrogenesis and bone ossification in zebrafish model mimicking CATSHL syndrome partially via enhanced Wnt/β-catenin signaling. Theranostics. 2020;10(16):7111–30. doi: 10.7150/thno.45286 32641982 PMC7330844

[37] Nicolas HA, Hua K, Quigley H, Ivare J, Tesson F, Akimenko M-A. A CRISPR/Cas9 zebrafish lamin A/C mutant model of muscular laminopathy. Dev Dyn. 2022;251(4):645–61. doi: 10.1002/dvdy.427 34599606

[38] Goldman JA, Kuzu G, Lee N, Karasik J, Gemberling M, Foglia MJ, et al. Resolving heart regeneration by replacement histone profiling. Dev Cell. 2017;40(4):392-404.e5. doi: 10.1016/j.devcel.2017.01.013 28245924 PMC5367476

[39] Dorey K, Amaya E. FGF signalling: diverse roles during early vertebrate embryogenesis. Development. 2010;137(22):3731–42. doi: 10.1242/dev.037689 20978071 PMC3747497

[40] Liu Z, Meng Y, Ishikura A, Kawakami A. Live tracking of basal stem cells of the epidermis during growth, homeostasis and injury response in zebrafish. Development. 2024;151(2):dev202315. doi: 10.1242/dev.202315 38265193

[41] Lee Y, Grill S, Sanchez A, Murphy-Ryan M, Poss KD. Fgf signaling instructs position-dependent growth rate during zebrafish fin regeneration. Development. 2005;132(23):5173–83. doi: 10.1242/dev.02101 16251209

[42] Grajevskaja V, Camerota D, Bellipanni G, Balciuniene J, Balciunas D. Analysis of a conditional gene trap reveals that tbx5a is required for heart regeneration in zebrafish. PLoS One. 2018;13(6):e0197293. doi: 10.1371/journal.pone.0197293 29933372 PMC6014646

[43] Bangru S, Diegmiller R, Di Talia S, Poss KD. Signal control during tissue regeneration in adult animals. Nat Rev Mol Cell Biol. 2026;27(4):316–35. doi: 10.1038/s41580-025-00917-1 41219473 PMC12822818

[44] Peters B, Kirfel J, Büssow H, Vidal M, Magin TM. Complete cytolysis and neonatal lethality in keratin 5 knockout mice reveal its fundamental role in skin integrity and in epidermolysis bullosa simplex. Mol Biol Cell. 2001;12(6):1775–89. doi: 10.1091/mbc.12.6.1775 11408584 PMC37340

[45] Félix M-A, Barkoulas M. Pervasive robustness in biological systems. Nat Rev Genet. 2015;16(8):483–96. doi: 10.1038/nrg3949 26184598

[46] de Bruijn E, Cuppen E, Feitsma H. Highly Efficient ENU Mutagenesis in Zebrafish. Methods Mol Biol. 2009;546:3–12. doi: 10.1007/978-1-60327-977-2_1 19378094

[47] Trevarrow B. Techniques for optimizing the creation of mutations in zebrafish using N-ethyl-N-nitrosourea. Lab Anim (NY). 2011;40(11):353–61. doi: 10.1038/laban1111-353 22012195

[48] Riley BB, Grunwald DJ. Efficient induction of point mutations allowing recovery of specific locus mutations in zebrafish. Proc Natl Acad Sci U S A. 1995;92(13):5997–6001. doi: 10.1073/pnas.92.13.5997 7597068 PMC41629

