# Supplementary material for: Regeneration programs buffer genetic defects in animal development
Source: PLoS Biol. 2026 Sep 21;24(9):e3004011. doi: 10.1371/journal.pbio.3004011 (PMC13630312; doi:10.1371/journal.pbio.3004011)
Supplement: Supplementary file 1 [file pbio.3004011.s011.docx]

**Responses to Reviewer Comments:**

We are grateful to the reviewers for their enthusiasm and helpful critiques. Please find our responses below.

Rev. 1: Alejandro Sánchez Alvarado – note that this reviewer has signed the review
This manuscript by Ando et al. explores a fundamental and, in my opinion, long-underserved question in developmental and regenerative biology: Can the same genetic programs that orchestrate tissue regeneration also act in a surveillance or compensation role for genetic developmental defects?

The manuscript presents an innovative genetic analysis showing that regeneration-associated gene programs in zebrafish larvae can be triggered by developmental genetic defects, not just by injury. The work is methodologically rigorous and offers conceptual insight into buffering mechanisms during development.

However, the findings are currently restricted to the larval stage, where developmental plasticity is high and regeneration capacity is near maximal. Adult vertebrate tissues, including those in killifish and zebrafish, present additional regulatory constraints and often require overt injury to activate regeneration gene expression. Thus, it remains unclear whether the same genetic perturbations would elicit comparable regenerative responses in adult animals, where cellular context, positional information, and ECM signaling play much more prominent roles.

To strengthen the manuscript, the authors should explicitly acknowledge these limitations. Specifically, they should clarify that larval plasticity may underlie the observed activation of regeneration programs and that the results may not generalize to adult stages. This distinction will help contextualize the impact of their findings and encourage follow-up studies extending these concepts to mature tissues.

Strengths:
* Innovative Approach: The authors have executed an elegant genetic screen in zebrafish larvae that expands our understanding of how regeneration programs may be activated—by developmental genetic defects and not solely by injury.
* Experimental Rigor: The work is methodologically sound, employing both ENU mutagenesis and CRISPR/Cas9 validation, with well-chosen controls and convincing phenotypic analyses.
* Conceptual Advance: By showing that congenital defects can recruit regeneration-associated gene expression, the authors present a provocative model with implications for developmental robustness and organismal fitness.

Weaknesses and Limitations that MUST be addressed in a revised version:

* Developmental Restriction: The findings are limited to the larval stage, a period marked by exceptional developmental plasticity. It remains uncertain whether adult tissues, where regeneration capacity and regulatory mechanisms differ profoundly, would behave similarly in response to genetic injury.
* Generalization to Adults: The authors do not directly address whether the regenerative gene activation observed would persist in adult vertebrates, where positional cues, ECM signaling, and tissue context are more constrained.
* Acknowledgement of Plasticity: The manuscript would benefit from a stronger acknowledgement that larval plasticity may underlie these responses, and that such mechanisms may not operate in mature tissues.

A. We agree and have added the following text to the Discussion on page 7, paragraph 2 to address these points: “Many but not all adult vertebrate tissues activate and require regeneration programs to replace day to day cell loss or wear and tear. We have not tested how genetic lesions induced at the adult stage can be cloaked by regeneration programs, and we acknowledge that adult tissues may operate differently versus those undergoing initial development. Using conditional mutations or those inducing delayed-onset or progressive phenotypes could test this concept in mature tissues.”

Minor comments that if addressed would strengthen the paper:
1. Upstream Sensing: While the demonstration that enhancer activity responds to genetic defects is compelling, the nature of the signals that communicate tissue instability to regeneration-responsive enhancers remains mysterious. Is this damage-sensing a generalized feature of 'fragile epithelia' or a more gene-specific consequence in fras1 mutants? Single-cell analyses or pathway dissection here would move this from a stimulating observation to a mechanistic advance.

We agree that identifying the upstream signals that link congenital tissue instability to regeneration-responsive enhancers is an important open question. Our study was designed to establish the existence and generality of this phenomenon rather than to fully resolve the sensing mechanism. Key observations that argue against a *fras1*-specific effect are: *fgf20a* induction is observed across multiple genetically unrelated perturbations that disrupt epithelial or ECM integrity; and multiple independent injury-responsive TREEs are activated in *fras1* mutants. In the revised manuscript, we have included new RNA sequencing data indicating adoption of transcriptional signatures similar to amputation-induced regeneration (page 5, paragraph 3, and Figure 3E-H). These suggest engagement of a broader regeneration-linked regulatory state. The revised Discussion explicitly acknowledges the need to identify upstream signals (page 7, paragraph 2), and it outlines testable models whereby mutations causing ECM-associated mechanical stress, inflammation associated with barrier failure, or factors released from dying cells may converge on regeneration-responsive enhancers.

2. Functional Consequence: The finding that modulation of Fgf signaling exacerbates phenotypes in fras1 mutants is critical and well executed. However, I urge the authors to probe more deeply the physiological benefit imparted by regeneration programming in the context of developmental defects. Do these animals actually experience improved survival, function, or fitness as a result?

We agree that demonstrating physiological benefit is important. To reiterate, we propose that regeneration programs act as buffering mechanisms, and they might limit degeneration rather than fully restoring normal morphology. We directly tested whether regeneration-associated signaling limits the severity of congenital degeneration. We had included in the original submission that we were unable to usefully test if gain of function of Fgf signaling improves the outcome in *fras1* mutants, as inducing *fgf20a* even conditionally in larval fin fold epithelia reduced survival. *fras1* mutants die after their yolks are exhausted, and the exacerbated phenotype caused by blocking Fgf signaling does not substantially change survival. We have probed this more deeply by including a new experiment in Figure S5 and described on page 6, paragraph 3, with different conditions of Fgf blockade, involving quantitative analysis of the severity of degeneration.

3. Quantitative Strengthening: The manuscript would be aided by additional quantified data, both for phenotypic penetrance across lines and for the degree of enhancer activation. Figures could be more visually accessible, enabling readers to fully appreciate the statistical rigor the authors have clearly invested in their analyses.

We agree and have strengthened the quantitative presentation throughout. These include a new Figure S1B panel indicating the phenotypic penetrance in other candidate ENU mutant families. We provide quantitative measurements of degeneration of fin fold tissue in Figure S2A and Figure S3E, with descriptions in the text on page 3, paragraph 2, and page 5, paragraph 1, respectively. We provide a measures of TREE reporter activation in Figure S2B, Figure 3D, and Figure S3F. We also improved figure accessibility by adding higher-magnification insets, annotated features, and consistent scale bars/orientations. We hope these additions make the rigor of this work more apparent to readers.

Recommendation:
This is an important and stimulating piece of work. To maximize its impact and clarity, the authors should explicitly discuss the developmental limitations raised above and avoid overgeneralizing their conclusions to adult stages.

Rev. 2:
The manuscript by the authors, Ando K. et al., entitled "Buffering of Genetic Defects in Animal Development by Regeneration Programs", present evidence supporting a novel concept: the regenerative program is not limited to injury response but also functions as a surveillance mechanism that continuously monitors tissue integrity during embryonic development. To investigate this, they established a transgenic zebrafish enhancer trap line, fgf20a:EGFP (Green Fluorescent Protein). Using the classic chemical mutagen ENU, they treated 11 male fish, generated 460 F3 families, and screened about 10,000 young fish. They identified a mutant family ("budlight") carrying a premature stop codon (Y3708*) in fras1, confirmed by CRISPR knockout. To confirm this finding, they used CRISPR to knock out fras1, which fully reproduced the phenotype (green fluorescence), validating the role of fras1. Furthermore, they selected 15 genes homologous to human developmental disease genes, knocked each out using CRISPR/Cas9, and observed whether the reporter could be induced. Attempts to overexpress fgf20a under the epithelial-specific itgb4 promoter caused embryonic lethality during gastrulation. Conversely, blocking Fgf signaling with heat-shock inducible dnFgfr1 exacerbated tail fin degeneration in fras1 mutants and increased posterior body twisting, suggesting that endogenous Fgf signaling indeed provides a protective function.

I have the following comments on this manuscript:
1. I think the new concept is quite interesting, but the author should clearly define what constitutes the "regeneration program". As the authors would agree, genes and pathways essential for tissue regeneration also play an important role in early embryonic development. The observed phenotypes may involve feedback loops rather than a distinct surveillance mechanism.

We agree that the term “regeneration program” might mean different things to different people. In our definition, this refers broadly to changes in gene expression that occur during regeneration versus what would normally be in place in the absence of injury. We now state this in the Introduction on page 2, paragraph 1. Moreover, we have now included transcriptomics data (page 5, paragraphs 3-5; Figures 3E-H and S4) comparing tail tissue under a standard larval tail regeneration paradigm compared with uninjured WT tissue. By the definition above, these transcriptional changes induced after tail amputation and associated with regeneration constitute the regeneration program for this context. We then compared this to the *fras1* mutant fin fold transcriptome at 4 dpf. We find that genes induced after tail amputation versus uninjured tails are significantly enriched among genes with higher levels in *fras1* mutant tails versus uninjured tails. These analyses support the conclusion that congenital epithelial instability in *fras1* mutants engages a regeneration-linked transcriptional program that overlaps substantially with canonical injury-induced regeneration, rather than reflecting a generic developmental feedback loop.

2. Was functional repair actually achieved after the induction of the regeneration program, and was mortality reduced? Whether the chemical treatment can also induce the activation of the regeneration program. These points are critical to support the proposed buffering role.

We agree that demonstrating physiological benefit is important. To reiterate, we propose that regeneration programs act as buffering mechanisms, and they might limit degeneration rather than fully restoring normal morphology. We directly tested whether regeneration-associated signaling limits the severity of congenital degeneration. We had included in the original submission that we were unable to usefully test if gain of function of Fgf signaling improves the outcome in *fras1* mutants, as inducing *fgf20a* even conditionally in larval fin fold epithelia reduced survival. *fras1* mutants die after their yolks are exhausted, and the exacerbated phenotype caused by blocking Fgf signaling does not substantially change survival. We have probed this more deeply by including a new experiment in Figure S5 and described on page 6, paragraph 3, with different conditions of Fgf blockade, involving quantitative analysis of the severity of degeneration. With regard to chemical treatment and its effects on regeneration programs, we agree this could be useful in testing our concept. Because many compounds can be toxic during larval development, we did not undertake such a screen in this study.

3. I am not convinced by the F0 assay shown in Fig. 3. The injection of Cas9 protein and sgRNA can cause developmental defects, and the craniofacial structure is particularly fragile. Therefore, the phenotype observed in the F0 assay may not accurately reflect the targeted gene disruption and should be interpreted with caution.

We agree that F_0_ CRISPR assays can be confounded by injection-associated toxicity, off-target effects and mosaicism, and these factors can be particularly relevant in craniofacial tissues. We have therefore strengthened the experimental controls and clarified the interpretation of experiments in Fig. 3. First, we added Cas9-only and non-targeting sgRNA injection controls, scored in parallel, which show minimal background reporter activation and fin fold degeneration. This is shown in Figure S3B-D and mentioned on page 4, paragraph 6.

4. The experiments are limited to zebrafish fins and craniofacial structure. Does the same principle apply to other organs (brain, heart, kidney)? Expanding or discussing this limitation would strengthen the manuscript.

Our experiments primarily focus on larval fin fold, due to the fact that this concept arose from a large-scale ENU mutagenesis screen that assessed responses to fin fold amputation. We have revised the Discussion on page 6, paragraph 5, to mention the caveat that whether a similar concept applies to internal organs (e.g., heart, kidney, brain) remains to be determined. We highlight that fin fold provides a sensitive and quantitative sentinel tissue for testing the principle due to its sensitivity to defects in epithelial integrity, the availability of a canonical amputation-based regeneration benchmark, and the external nature that can be qualitatively or quantitatively assessed in a straightforward manner.

5. The upstream trigger remains unclear. Is it membrane tension, ROS, immune factors, or another signal? The mechanism is currently a "black box." The authors should elaborate on potential pathways in the Discussion.

We agree that identifying the upstream signals that link congenital tissue instability to regeneration-responsive enhancers is an important open question. Our study was designed to establish the existence and generality of this phenomenon rather than to fully resolve the sensing mechanism. In the revised manuscript, we have included new RNA sequencing data indicating adoption of transcriptional signatures similar to amputation-induced regeneration (page 5, paragraphs 3-5; Figures 3E-H and S4). These suggest engagement of a broader regeneration-linked regulatory state. The revised Discussion explicitly acknowledges the need to identify upstream signals (page 7, paragraph 2), and it outlines testable models whereby mutations causing ECM-associated mechanical stress, inflammation associated with barrier failure, or factors released from dying cells may converge on regeneration-responsive enhancers.

Rev. 3:
The authors identified a mutant budlight through a screen that involves using a Tissue Regeneration Enhancer Element (TREE) for fgf20a transcription. In addition to activation of the TREE-controlled GFP, this mutant displays a degenerating finfold phenotype. The authors map the mutation to fraser extracellular matrix complex subunit 1 (fras1), which encodes a transmembrane protein that harbors von Willebrand factor-C and furin-like repeats that maintain adhesion between epithelial cells and mesenchymal cells. This gene is linked to human disorders: blistering, etc. The authors show that that fras1 is expressed in the epithelial cells of the fin folds, which supports their conclusion that it is responsible for the phenotype.

The authors use this mutant to generate the main message of the manuscript, which is regeneration is a way for organisms to buffer damage from congenital defects. They authors generate additional evidence to support this hypothesis by generating CRISPR mutants of other genes that produce finfold degeneration phenotypes to show that these mutants also induce the TREE-regulated expression of the gene trap locus (Fig. 3). In addition, the authors show that other injury-responsive TREEs are also activated in fras1 mutants (Fig. 4). Lastly, the authors provide evidence that inhibition of FGF signaling using a dominant-negative Fgf receptor 1 transgene exacerbates the degeneration phenotype, indicating the necessity of Fgf signaling to limit the degenerative effects of the mutant fras1 transcript (Fig. 4).

The authors provide evidence to support the claims that the injury from the congenital defects elicits a regeneration-like response, but I feel that this evidence can be strengthened. The one weakness of the manuscript is that it does not provide any mechanism for how the zebrafish is inducing regeneration in response to congenital defects, which the authors point out in the second paragraph of their discussion section. Therefore, the manuscript needs to be very clear on the novelty of the concept that regeneration is specifically activated in regeneration contexts to ameliorate congenital defects. In this regard, I have several points:

1. Considering there is so much literature about the benefit of stem and progenitor cells to regenerate tissues and counter damage from congenital defects either by implantation or facilitating the body's on stem cell populations, how does the authors' message that regeneration is a means to ameliorate damage from congenital defects differ from the established concept of the benefits of endogenous stem cell pools as a means of compensating for injures from congenital defects?

We agree that it is established that endogenous stem or progenitor populations that can contribute to cell loss by injury, day-to-day loss, or degeneration. Our study does not challenge this concept, but instead addresses a distinct question: whether canonical injury-responsive regeneration programs themselves can be activated by mutation-induced developmental tissue instability in the absence of overt injury. Here, we demonstrate that injury-responsive enhancer elements and a transcriptional program empirically defined by posterior tissue amputation are activated in early, developing animals by congenital tissue instability in the absence of overt injury. We therefore propose that regeneration programs also constitute a context-dependent regulatory state that can be engaged to buffer genetic defects in development. We see this as a new way to think about the utility of regeneration programs and how they may be targets for natural selection. We provide this explanation in an expanded Discussion.

2. Several of the genes that the authors examined are in the endochondral skeleton, which is a tissue not found in the finfold. What exactly are the "malformed skeletal elements"? The authors should also provide more descriptive details about the different "degeneration" phenotypes and provide higher magnified images to visualize much better the degeneration phenotypes and the changes in the expression of the TREE reporter.

We agree that greater anatomical precision and phenotypic clarity are needed. We have revised the text to explicitly specify the affected structures rather than using the general term “malformed skeletal elements.” We also clarify that TREE activation was assessed in the fin fold epithelium, whereas skeletal phenotypes are referenced to confirm effective gene disruption and contextualize the congenital defects. To improve clarity, we have provided higher-quality, annotated images of fin fold degeneration in Figures 2 and S3, and we have provided quantifications for degeneration severity and TREE activation (Figure S3E, F).

3. To assess whether the regenerating program is truly activated in the tissues that manifest human congenital defects, the authors should assess TREE activation in the specific tissues in which the congenital defects are known to manifest (endochondral skeleton, heart, etc.) and not just the finfold. Otherwise, the authors should provide good arguments for why assessing only the finfold suffices.

Our experiments primarily focus on larval fin fold, due to the fact that this concept arose from a large-scale ENU mutagenesis screen that assessed responses to fin fold amputation. We have revised the Discussion on page 6, paragraph 5, to mention the caveat that whether a similar concept applies to internal organs (e.g., heart, kidney, brain) remains to be determined. We highlight that fin fold provides a sensitive and quantitative sentinel tissue for testing the principle due to its sensitivity to defects in epithelial integrity, the availability of a canonical amputation-based regeneration benchmark, and the external nature that can be qualitatively or quantitatively assessed in a straightforward manner.

4. If the authors are concluding that the regeneration program is active, then the authors should show the genuine activation of a broader panel of regeneration genes by qRT-PCR/in situ hybridization, for example genes that represent several major developmental gene pathways: Wnt, BMP, Shh as examples. It is possible that only fgf20a is activated (although the expression of fgf20a was never directly assessed, since the GFP is directly controlled by the stress-induced hsp70l promoter), which may not reflect a genuine regeneration response. If only fgf20a is activated, one could argue that it may just reflect a pro-survival mechanism that limits cell death.

We are grateful for this comment. We have now included transcriptomics data (Figures 3E-H and S4) comparing tail tissue under a standard larval tail regeneration paradigm compared with uninjured WT tissue. By the definition above, these transcriptional changes induced after tail amputation and associated with regeneration constitute the regeneration program for this context. We then compared this to the *fras1* mutant fin fold transcriptome at 4 dpf. We find that genes induced after tail amputation versus uninjured tails are significantly enriched among genes with higher levels in *fras1* mutant tails versus uninjured tails. These analyses support the conclusion that congenital epithelial instability in *fras1* mutants engages a broad, regeneration-linked transcriptional program that overlaps substantially with canonical injury-induced regeneration as we now state on page 6, paragraph 1.

Also, we have added qRT-PCR based quantification in Figure 1F and page 3, paragraph 2, which indicates that *fgf20a* gene expression is sharply induced in *budlight*/*fras1* mutants; thus, the gene reporter presence is not simply reporting activation of the *hsp70* promoter.

5. In the discussion section, paragraph one, the authors suggest that regeneration mechanisms are retained for countering defects during morphogenesis as well as suggest that this is the reason why some vertebrates (such as zebrafish) have conserved their regeneration programs. Considering the several genes chosen cause human congenital defects, couldn't humans have retained some regeneration mechanisms during early life stages (embryo, pre-juvenile, juvenile) as observed in zebrafish? The authors should provide more discussion on this point using examples in which human tissues regenerate to ameliorate disease phenotypes (e.g. skin using its stem cell population, muscle using its satellite cell population, etc.) at least during early stages of the diseases mentioned in this manuscript.

We agree and have expanded the Discussion to address this point explicitly. While most adult human tissues exhibit limited regenerative capacity, multiple tissues retain regeneration capacity including skin, skeletal muscle, liver, blood and intestinal epithelium. We now discuss on page 7, paragraph 3, how our findings are likely relevant to certain human disease contexts – not only Fraser Syndrome, but others like Duchenne’s muscular dystrophy, during which highly regenerative tissues experience losses in cells or tissue integrity.
