# Supplementary material for: Regeneration programs buffer genetic defects in animal development
Source: PLoS Biol. 2026 Sep 21;24(9):e3004011. doi: 10.1371/journal.pbio.3004011 (PMC13630312; doi:10.1371/journal.pbio.3004011)
Supplement: Supplementary file 2 [file pbio.3004011.s012.docx]

August 13, 2026

Ines Alvarez-Garcia, PhD

Senior Editor

*PLOS Biology*

[ialvarez-garcia@plos.org](mailto:ialvarez-garcia@plos.org)

Dear Ines,

Thank you for your email of July 14 with 3 final comments and suggestions from Reviewer 3. We address these comments below and in the second revised version of our manuscript. Additionally, we have organized all data related to this manuscript in folders and submitted these for consideration by Dryad, with the accession number provided in the Data Availability Statement” in our manuscript.

**Reviewer 3:**

The proposed hypothesis that regeneration responses are activated by genetic perturbations that generate congenital defects and may explain differences in expressivity and penetrance is important. The data using the zebrafish larva is convincing and supports the authors conclusions that a meaning part of the regeneration program is activated in response to targeting genes associated with human congenital defects. However, the question remains does this occur in clinical models that reproduce the congenital defects, which is an important point of this manuscript. The authors provide supporting arguments from the literature that humans (clinical aspect of congenital defects) do have organs and organ systems that have regeneration capacity, but the brief descriptions of the supporting information do not provide direct evidence that a congenital defect is ameliorated/compensated for by an activated "broader regenerative response" and not just continuous homeostatic maintenance (e.g. the continual generation of skin from the basal stem cells).

In this regard, my original point 3 applies: "To assess whether the regenerating program is truly activated in the tissues that manifest human congenital defects, the authors should assess TREE activation in the specific tissues in which the congenital defects are known to manifest (endochondral skeleton, heart, etc.) and not just the finfold. Otherwise, the authors should provide good arguments for why assessing only the finfold suffices." The authors addressed this point with an explanation for how their hypothesis came to them and with a description about the ease in characterizing the larval regeneration response of the finfold. However, they did not provide an assessment (their own experiment or data from the literature) on whether there is a regeneration response to the known clinically related (e.g. mouse model) contexts of the Fraser syndrome congenital defect or the other human-disease genes they used to show Fgf20a-TREE activation in figure 2. If there isn't citable experimental data in the literature that directly applies to the authors hypothesis, it is likely that there is deep-sequencing data from one or more of the clinical models of the congenital-defect genes that the authors tested in larval fish. How much of regeneration response of the zebrafish larva is similar to the expression data from the clinical model? One possible comparison would be the tbx5 defects: transcriptomic analyses were performed in fish and mouse embryos, so such a comparison with existing data should be possible. The more similarities in the transcriptional responses between the fish and the clinical model, the more the authors can claim that mutation-induced tissue instabilities of congenital defects lead to (are compensated by) activated regeneration responses. As shown in figure 3, If comparable data in the literature can be found, the authors have the ability to make these comparisons, so it should only require a minor revision.

**A.** We thank the reviewer for pressing this point, and we accept the distinction being drawn between a genuinely activated regenerative response and ongoing homeostatic maintenance, and it is precisely this distinction that shapes our experimental design. We have researched this topic and feel it is best to say why we have not performed the proposed cross-species transcriptomic comparison, rather than to present an analysis that we do not think will be informative due to the data that are available. From the start we will suggest that transcript-level overlap is a supportive but suboptimal test of the activation of regeneration programs on its own, as the genes that constitute the injury response such as AP-1 factors, IL-6-family cytokines, matrix metalloproteinases, FGF ligands, matricellular proteins such as *Postn* are differentially regulated during development, inflammation, fibrosis, neoplasia and normal tissue turnover. Transcriptional profiling is therefore most informative when interpreted alongside an independent readout of regulatory state, and our study was built to provide exactly that pairing: the transcriptome helps define the program, while regeneration enhancer elements establish that it has been engaged. We built this study on TREE-engaged reporters since regeneration enhancer elements resolve this ambiguity: LEN, REN and the fgf20a regulatory sequences are transcriptionally quiet in intact larval fin fold and are activated specifically upon amputation (Kang et al. 2016; Fig. 4B). Their ectopic activation in uninjured fras1 mutants is therefore our best evidence that an injury-gated regulatory program has been deployed, which a gene-level expression overlap could not establish. Our transcriptomic and enhancer data are thus mutually reinforcing, drawn from the same tissue, lesion and animal. The comparison the reviewer proposes would remove that pairing. No mammalian counterparts of these enhancer elements have been characterized, so a cross-species analysis would rest on transcript overlap alone, without the regulatory readout that makes our own transcriptomic data interpretable and it would do so across datasets that differ in species, tissue and developmental stage.

The available datasets also have deficiencies that we do not think a careful analysis can overcome. The mammalian transcriptomic resources for the genes in Fig. 2 are largely cardiac, and tbx5, the reviewer's suggested test case, may be the least suitable of them, because tbx5a is itself required for the regenerative response: conditional inactivation in cardiomyocytes impairs zebrafish heart regeneration after ventricular resection [1]. A tbx5 lesion therefore compromises both the tissue and the machinery that would buffer it, which confuses the interpretation. We mention this in the second paragraph of the revised Discussion. For the genes whose lesions produce the tissue instability we model -- such as the Fraser complex, and the fin- and limb-associated genes-- deep sequencing data are largely absent. We could identify no transcriptomic data for the classic blebs mouse mutants, and the recently reported constitutive Frem2 knockout is characterized by histology only [2]. The single Fraser-complex dataset we located compares three Frem2 mutant embryos with one wild-type littermate at E13.5 in whole embryos; a null result from that comparison would not distinguish absent biology from absent power [3]. Available skin-fragility transcriptomes are postnatal or adult such as the RDEB model with patient-derived Col7a1 alleles, for instance, profiles two-week-old back skin, past the regenerative window and after repeated cycles of blistering and healing that might confound the primary lesion [4].

We acknowledge that our previous Discussion offered general statements where direct evidence was needed, and we have replaced them. The most informative mammalian precedent is one we had not previously cited. In keratin 5 (K5)-null mice, a germline mutation causing spontaneous epidermal cytolysis, there is induction of the wound-healing keratin K6 in the suprabasal epidermis specifically in cytolyzed areas; that is, a canonical injury-response gene induced in the absence of any experimental wound and spatially restricted to the regions undergoing degeneration [5]. We mention this in the 4^th^ paragraph of the revised Discussion. The comparison is internally controlled: K5-null mice lack basal keratin filaments and are more severely affected than K14-null mice, in which the same K6 response is not observed, so the injury program tracks the severity of tissue instability rather than the presence of a keratin mutation as such. We would add that our own transcriptomic dataset of uninjured fras1/budlight mutant, uninjured sibling, and 1 dpa amputated fin fold is itself a demonstration that a genetic lesion induces regeneration gene expression programs in unwounded tissue *in vivo*, and pairs that transcriptional evidence with enhancer activation in the same tissue. Additionally, from the perspective of the large community that works with zebrafish, ours is in fact a pre-clinical model of the phenomenon the reviewer requests, rather than as a stand-in example of a mammalian model awaiting confirmation. We have articulated claims to try to represent our data as accurately as possible. In summary, we state that our conclusions concern a vertebrate with especially high regenerative capacity, and that whether this mechanism operates in mammals is a principal question the work raises. We see this as a conservative response rather than leaning on a comparison built on datasets that may be mismatched versus our evidence assessing three independent, injury-gated elements at unlinked loci, activated in uninjured mutant tissue, with the response shown to be functionally load-bearing by dnFgfr1 induction.

In regards to my original second point (Point 2): The applicable clinical manifestation of Fra1/FRA1 gene defect is skin blistering. The resolution of the images of the skin phenotype are still not high enough to observed this. Do the authors observe blistering in the zebrafish larva skin as observed in clinical models? How much of the clinical phenotype of the skin are manifested in the larva? How do they think the regeneration response affects this?

**A.** We thank the reviewer and agree that the previous images did not adequately illustrate the epithelial separation phenotype. We have replaced those panels with higher-resolution images highlighting the affected regions (Supplementary Figure 2C). These show discrete regions of epidermal detachment in the caudal fin fold and is reminiscent of the blistering seen in the fin fold of *pinfin* mutant [6]. Our model therefore reproduces the epithelial adhesion defect.

On the extent to which the clinical phenotype is manifested, the shared feature is the primary adhesion failure and its immediate consequence, sub-epidermal blistering. Notably, the blistering we observe is confined to 1–2 dpf and is not detected at later stages. We would emphasize that this transience is characteristic of the disorder rather than a divergence from it; in Fraser syndrome, the pathogenic events are transient embryonic epidermal blisters whose formation and subsequent resolution leave the residual defects that constitute the clinical syndrome. On how the regeneration response relates to this, we want to clarify what our experiments can and cannot address. Our dnFgfr1 induction begins at 2 dpf and later, whereas the blistering window is 1–2 dpf. The two are therefore temporally separated by design, and our data cannot speak to whether Fgf-dependent signaling prevents or resolves the initial epithelial separation. What the dnFgfr1 experiments test is the consequence of blunting the response after tissue instability has been established, and there the effect is clear: degeneration and posterior distortion are exacerbated in mutants relative to heat-shocked wild-type and heterozygous controls (Fig. 4G–I). Our claim is accordingly that the regeneration-associated program is engaged downstream of, and in response to, the tissue instability produced by the genetic lesion, and that it limits the resulting degeneration but not that it prevents the initiating blistering event.

In Figure 3H, it seems as though there are relatively few categories for such a large number of gene changes. It is also unclear why there is so little overlap among these categories. The authors should provide a supplemental list of the genes that have changed and should organize this list to the categories they described.

**A.** We thank the reviewer for this suggestion. We agree that the original figure did not sufficiently explain the relationship between the displayed biological categories and the underlying differentially expressed genes. We have therefore added a new Supplementary Table 3 listing all differentially expressed genes together with fold change, adjusted P value and the biological categories identified from GO analysis. We have also expanded the Figure 3H legend to explain that the displayed categories represent representative nonredundant functional groups selected after collapsing highly overlapping ontology terms. The limited overlap among displayed categories therefore reflects the visualization strategy rather than an absence of shared biological processes.

Thank you for all your help with our manuscript.

Sincerely,

Ken Poss

**References**

1.	Grajevskaja V, Camerota D, Bellipanni G, Balciuniene J, Balciunas D. Analysis of a conditional gene trap reveals that tbx5a is required for heart regeneration in zebrafish. PLoS One. 2018;13(6):e0197293. Epub 20180622. doi: 10.1371/journal.pone.0197293. PubMed PMID: 29933372; PubMed Central PMCID: PMCPMC6014646.

2.	Simikyan RG, Zhang X, Strelkova O, Li N, Zhu M, Eckhard A, et al. Frem2 knockout mice exhibit Fraser syndrome phenotypes and neonatal lethality due to bilateral renal agenesis. Sci Rep. 2025;15(1):32956. Epub 20250926. doi: 10.1038/s41598-025-14737-y. PubMed PMID: 41006360; PubMed Central PMCID: PMCPMC12475133.

3.	Zhang X, Wang R, Wang T, Zhang X, Dongye M, Wang D, et al. The Metabolic Reprogramming of Frem2 Mutant Mice Embryos in Cryptophthalmos Development. Front Cell Dev Biol. 2020;8:625492. Epub 20210108. doi: 10.3389/fcell.2020.625492. PubMed PMID: 33490088; PubMed Central PMCID: PMCPMC7820765.

4.	Takaki S, Shimbo T, Ikegami K, Kitayama T, Yamamoto Y, Yamazaki S, et al. Generation of a recessive dystrophic epidermolysis bullosa mouse model with patient-derived compound heterozygous mutations. Lab Invest. 2022;102(6):574-80. Epub 20220212. doi: 10.1038/s41374-022-00735-5. PubMed PMID: 35152273.

5.	Peters B, Kirfel J, Bussow H, Vidal M, Magin TM. Complete cytolysis and neonatal lethality in keratin 5 knockout mice reveal its fundamental role in skin integrity and in epidermolysis bullosa simplex. Mol Biol Cell. 2001;12(6):1775-89. doi: 10.1091/mbc.12.6.1775. PubMed PMID: 11408584; PubMed Central PMCID: PMCPMC37340.

6.	Carney TJ, Feitosa NM, Sonntag C, Slanchev K, Kluger J, Kiyozumi D, et al. Genetic analysis of fin development in zebrafish identifies furin and hemicentin1 as potential novel fraser syndrome disease genes. PLoS Genet. 2010;6(4):e1000907. Epub 20100415. doi: 10.1371/journal.pgen.1000907. PubMed PMID: 20419147; PubMed Central PMCID: PMCPMC2855323.
