# Supplementary material for: Regeneration programs buffer genetic defects in animal development
Source: PLoS Biol. 2026 Sep 21;24(9):e3004011. doi: 10.1371/journal.pbio.3004011 (PMC13630312; doi:10.1371/journal.pbio.3004011)
Supplement: Peer review history

# Editor report: Decision Letter 0

**DOI:** 10.1371/journal.pbio.3004011.r001

**Authors:** Ines Alvarez-Garcia

7 Nov 2025

Dear Dr Poss,

Thank you for submitting your manuscript entitled "Buffering of genetic defects in animal development by regeneration programs" for consideration as a Short Reports by PLOS Biology.

Your manuscript has now been evaluated by the PLOS Biology editorial staff as well as by an academic editor with relevant expertise and I am writing to let you know that we would like to send your submission out for external peer review.

However, before we can send your manuscript to reviewers, we need you to complete your submission by providing the metadata that is required for full assessment. To this end, please login to Editorial Manager where you will find the paper in the 'Submissions Needing Revisions' folder on your homepage. Please click 'Revise Submission' from the Action Links and complete all additional questions in the submission questionnaire.

Once your full submission is complete, your paper will undergo a series of checks in preparation for peer review. After your manuscript has passed the checks it will be sent out for review. To provide the metadata for your submission, please Login to Editorial Manager (https://www.editorialmanager.com/pbiology) within two working days, i.e. by Nov 10 2025 11:59PM.

If your manuscript has been previously peer-reviewed at another journal, PLOS Biology is willing to work with those reviews in order to avoid re-starting the process. Submission of the previous reviews is entirely optional and our ability to use them effectively will depend on the willingness of the previous journal to confirm the content of the reports and share the reviewer identities. Please note that we reserve the right to invite additional reviewers if we consider that additional/independent reviewers are needed, although we aim to avoid this as far as possible. In our experience, working with previous reviews does save time.

If you would like us to consider previous reviewer reports, please edit your cover letter to let us know and include the name of the journal where the work was previously considered and the manuscript ID it was given. In addition, please upload a response to the reviews as a 'Prior Peer Review' file type, which should include the reports in full and a point-by-point reply detailing how you have or plan to address the reviewers' concerns.

During the process of completing your manuscript submission, you will be invited to opt-in to posting your pre-review manuscript as a bioRxiv preprint. Visit http://journals.plos.org/plosbiology/s/preprints for full details. If you consent to posting your current manuscript as a preprint, please upload a single Preprint PDF.

Feel free to email us at plosbiology@plos.org if you have any queries relating to your submission.

Kind regards,

Ines

Ines

--

Ines Alvarez-Garcia, PhD

Senior Editor

PLOS Biology

ialvarez-garcia@plos.org

# Peer review: Decision Letter 1

**DOI:** 10.1371/journal.pbio.3004011.r002

**Authors:** Ines Alvarez-Garcia

19 Dec 2025

Dear Dr Poss,

Thank you for your patience while your manuscript entitled "Buffering of genetic defects in animal development by regeneration programs" was peer-reviewed at PLOS Biology. The manuscript has now been evaluated by the PLOS Biology editors, an Academic Editor with relevant expertise, and by three independent reviewers.

As you will see, the reviewers find the conclusions interesting and worth pursuing, but they also raise several issues that would need to be addressed before we can consider the manuscript for publication. Reviewer 1 thinks that the findings are restricted to the larval stage, in which developmental plasticity is high along with the regeneration capacity, thus the limitations should be acknowledged stating clarily that larval plasticity may underlie the observed activation of regeneration programs, and that the results do not apply to adult stages. Reviewer 2 notes that the ‘regeneration program’ should be clearly defined and also whether or not functional repair is achieved after the induction of regeneration, among other issues. Reviewer 3 asks to assess TREE activation in the specific tissues in which the congenital defects are known to manifest and not just the finfold – or discuss why assessing only this is enough – and that the activation of a broader panel of regeneration genes should be shown by qRT-PCR or in situ hybridisation.

In light of the reviews, we would like to invite you to revise the work to thoroughly address the reviewers' reports. Given the extent of revision needed, we cannot make a decision about publication until we have seen the revised manuscript and your response to the reviewers' comments. Your revised manuscript is likely to be sent for further evaluation by all or a subset of the reviewers.

In addition to these revisions, you will need to complete some formatting changes, which you will receive in a follow up email. A member of our team will be in touch with a set of requests shortly.

We expect to receive your revised manuscript within 3 months. Please email us (plosbiology@plos.org) if you have any questions or concerns, or would like to request an extension.

At this stage, your manuscript remains formally under active consideration at our journal; please notify us by email if you do not intend to submit a revision so that we may withdraw it.

**IMPORTANT - SUBMITTING YOUR REVISION**

Your revisions should address the specific points made by each reviewer. Please submit the following files along with your revised manuscript:

1. A 'Response to Reviewers' file - this should detail your responses to the editorial requests, present a point-by-point response to all of the reviewers' comments, and indicate the changes made to the manuscript.

*NOTE: In your point-by-point response to the reviewers, please provide the full context of each review. Do not selectively quote paragraphs or sentences to reply to. The entire set of reviewer comments should be present in full and each specific point should be responded to individually, point by point.

You should also cite any additional relevant literature that has been published since the original submission and mention any additional citations in your response.

2. In addition to a clean copy of the manuscript, please also upload a 'track-changes' version of your manuscript that specifies the edits made. This should be uploaded as a "Revised Article with Changes Highlighted" file type.

3. Resubmission Checklist

When you are ready to resubmit your revised manuscript, please refer to this resubmission checklist: https://plos.io/Biology_Checklist

To submit a revised version of your manuscript, please go to https://www.editorialmanager.com/pbiology/ and log in as an Author. Click the link labelled 'Submissions Needing Revision' where you will find your submission record.

Please make sure to read the following important policies and guidelines while preparing your revision and fulfil the editorial requests:

a) *PLOS Data Policy*

Please note that as a condition of publication PLOS' data policy (http://journals.plos.org/plosbiology/s/data-availability) requires that you make available all data used to draw the conclusions arrived at in your manuscript. If you have not already done so, you must include any data used in your manuscript either in appropriate repositories, within the body of the manuscript, or as supporting information (N.B. this includes any numerical values that were used to generate graphs, histograms etc.). Please also indicate in each figure legend where the data can be found. For an example see here: http://www.plosbiology.org/article/info%3Adoi%2F10.1371%2Fjournal.pbio.1001908#s5

b) *Published Peer Review*

Please note while forming your response, if your article is accepted, you may have the opportunity to make the peer review history publicly available. The record will include editor decision letters (with reviews) and your responses to reviewer comments. If eligible, we will contact you to opt in or out. Please see here for more details:

https://blogs.plos.org/plos/2019/05/plos-journals-now-open-for-published-peer-review/

c) *Blot and Gel Data Policy*

Please provide the original, uncropped and minimally adjusted images supporting all blot and gel results reported in an article's figures or Supporting Information files. We will require these files before a manuscript can be accepted so please prepare them now, if you have not already uploaded them. Please carefully read our guidelines for how to prepare and upload this data: https://journals.plos.org/plosbiology/s/figures#loc-blot-and-gel-reporting-requirements

d) *Protocols deposition*

To enhance the reproducibility of your results, we recommend that if applicable you deposit your laboratory protocols in protocols.io, where a protocol can be assigned its own identifier (DOI) such that it can be cited independently in the future. For instructions see: https://journals.plos.org/plosbiology/s/submission-guidelines#loc-materials-and-methods

Thank you again for your submission to our journal. We hope that our editorial process has been constructive thus far, and we welcome your feedback at any time. Please don't hesitate to contact us if you have any questions or comments.

Sincerely,

Ines

--

Ines Alvarez-Garcia, PhD

Senior Editor

PLOS Biology

ialvarez-garcia@plos.org

------------------------------------

Reviewers' comments

Rev. 1: Alejandro Sánchez Alvarado – note that this reviewer has signed the review

This manuscript by Ando et al. explores a fundamental and, in my opinion, long-underserved question in developmental and regenerative biology: Can the same genetic programs that orchestrate tissue regeneration also act in a surveillance or compensation role for genetic developmental defects?

The manuscript presents an innovative genetic analysis showing that regeneration-associated gene programs in zebrafish larvae can be triggered by developmental genetic defects, not just by injury. The work is methodologically rigorous and offers conceptual insight into buffering mechanisms during development.

However, the findings are currently restricted to the larval stage, where developmental plasticity is high and regeneration capacity is near maximal. Adult vertebrate tissues, including those in killifish and zebrafish, present additional regulatory constraints and often require overt injury to activate regeneration gene expression. Thus, it remains unclear whether the same genetic perturbations would elicit comparable regenerative responses in adult animals, where cellular context, positional information, and ECM signaling play much more prominent roles.

To strengthen the manuscript, the authors should explicitly acknowledge these limitations. Specifically, they should clarify that larval plasticity may underlie the observed activation of regeneration programs and that the results may not generalize to adult stages. This distinction will help contextualize the impact of their findings and encourage follow-up studies extending these concepts to mature tissues.

Strengths:

* Innovative Approach: The authors have executed an elegant genetic screen in zebrafish larvae that expands our understanding of how regeneration programs may be activated—by developmental genetic defects and not solely by injury.

* Experimental Rigor: The work is methodologically sound, employing both ENU mutagenesis and CRISPR/Cas9 validation, with well-chosen controls and convincing phenotypic analyses.

* Conceptual Advance: By showing that congenital defects can recruit regeneration-associated gene expression, the authors present a provocative model with implications for developmental robustness and organismal fitness.

Weaknesses and Limitations that MUST be addressed in a revised version:

* Developmental Restriction: The findings are limited to the larval stage, a period marked by exceptional developmental plasticity. It remains uncertain whether adult tissues, where regeneration capacity and regulatory mechanisms differ profoundly, would behave similarly in response to genetic injury.

* Generalization to Adults: The authors do not directly address whether the regenerative gene activation observed would persist in adult vertebrates, where positional cues, ECM signaling, and tissue context are more constrained.

* Acknowledgement of Plasticity: The manuscript would benefit from a stronger acknowledgement that larval plasticity may underlie these responses, and that such mechanisms may not operate in mature tissues.

Minor comments that if addressed would strengthen the paper:

1. Upstream Sensing: While the demonstration that enhancer activity responds to genetic defects is compelling, the nature of the signals that communicate tissue instability to regeneration-responsive enhancers remains mysterious. Is this damage-sensing a generalized feature of 'fragile epithelia' or a more gene-specific consequence in fras1 mutants? Single-cell analyses or pathway dissection here would move this from a stimulating observation to a mechanistic advance.

2. Functional Consequence: The finding that modulation of Fgf signaling exacerbates phenotypes in fras1 mutants is critical and well executed. However, I urge the authors to probe more deeply the physiological benefit imparted by regeneration programming in the context of developmental defects. Do these animals actually experience improved survival, function, or fitness as a result?

3. Quantitative Strengthening: The manuscript would be aided by additional quantified data, both for phenotypic penetrance across lines and for the degree of enhancer activation. Figures could be more visually accessible, enabling readers to fully appreciate the statistical rigor the authors have clearly invested in their analyses.

Recommendation:

This is an important and stimulating piece of work. To maximize its impact and clarity, the authors should explicitly discuss the developmental limitations raised above and avoid overgeneralizing their conclusions to adult stages.

Rev. 2:

The manuscript by the authors, Ando K. et al., entitled "Buffering of Genetic Defects in Animal Development by Regeneration Programs", present evidence supporting a novel concept: the regenerative program is not limited to injury response but also functions as a surveillance mechanism that continuously monitors tissue integrity during embryonic development. To investigate this, they established a transgenic zebrafish enhancer trap line, fgf20a:EGFP (Green Fluorescent Protein). Using the classic chemical mutagen ENU, they treated 11 male fish, generated 460 F3 families, and screened about 10,000 young fish. They identified a mutant family ("budlight") carrying a premature stop codon (Y3708*) in fras1, confirmed by CRISPR knockout. To confirm this finding, they used CRISPR to knock out fras1, which fully reproduced the phenotype (green fluorescence), validating the role of fras1. Furthermore, they selected 15 genes homologous to human developmental disease genes, knocked each out using CRISPR/Cas9, and observed whether the reporter could be induced. Attempts to overexpress fgf20a under the epithelial-specific itgb4 promoter caused embryonic lethality during gastrulation. Conversely, blocking Fgf signaling with heat-shock inducible dnFgfr1 exacerbated tail fin degeneration in fras1 mutants and increased posterior body twisting, suggesting that endogenous Fgf signaling indeed provides a protective function.

I have the following comments on this manuscript:

1. I think the new concept is quite interesting, but the author should clearly define what constitutes the "regeneration program". As the authors would agree, genes and pathways essential for tissue regeneration also play an important role in early embryonic development. The observed phenotypes may involve feedback loops rather than a distinct surveillance mechanism.

2. Was functional repair actually achieved after the induction of the regeneration program, and was mortality reduced? Whether the chemical treatment can also induce the activation of the regeneration program. These points are critical to support the proposed buffering role.

3. I am not convinced by the F0 assay shown in Fig. 3. The injection of Cas9 protein and sgRNA can cause developmental defects, and the craniofacial structure is particularly fragile. Therefore, the phenotype observed in the F0 assay may not accurately reflect the targeted gene disruption and should be interpreted with caution.

4. The experiments are limited to zebrafish fins and craniofacial structure. Does the same principle apply to other organs (brain, heart, kidney)? Expanding or discussing this limitation would strengthen the manuscript.

5. The upstream trigger remains unclear. Is it membrane tension, ROS, immune factors, or another signal? The mechanism is currently a "black box." The authors should elaborate on potential pathways in the Discussion.

Rev. 3:

The authors identified a mutant budlight through a screen that involves using a Tissue Regeneration Enhancer Element (TREE) for fgf20a transcription. In addition to activation of the TREE-controlled GFP, this mutant displays a degenerating finfold phenotype. The authors map the mutation to fraser extracellular matrix complex subunit 1 (fras1), which encodes a transmembrane protein that harbors von Willebrand factor-C and furin-like repeats that maintain adhesion between epithelial cells and mesenchymal cells. This gene is linked to human disorders: blistering, etc. The authors show that that fras1 is expressed in the epithelial cells of the fin folds, which supports their conclusion that it is responsible for the phenotype.

The authors use this mutant to generate the main message of the manuscript, which is regeneration is a way for organisms to buffer damage from congenital defects. They authors generate additional evidence to support this hypothesis by generating CRISPR mutants of other genes that produce finfold degeneration phenotypes to show that these mutants also induce the TREE-regulated expression of the gene trap locus (Fig. 3). In addition, the authors show that other injury-responsive TREEs are also activated in fras1 mutants (Fig. 4). Lastly, the authors provide evidence that inhibition of FGF signaling using a dominant-negative Fgf receptor 1 transgene exacerbates the degeneration phenotype, indicating the necessity of Fgf signaling to limit the degenerative effects of the mutant fras1 transcript (Fig. 4).

The authors provide evidence to support the claims that the injury from the congenital defects elicits a regeneration-like response, but I feel that this evidence can be strengthened. The one weakness of the manuscript is that it does not provide any mechanism for how the zebrafish is inducing regeneration in response to congenital defects, which the authors point out in the second paragraph of their discussion section. Therefore, the manuscript needs to be very clear on the novelty of the concept that regeneration is specifically activated in regeneration contexts to ameliorate congenital defects. In this regard, I have several points:

1. Considering there is so much literature about the benefit of stem and progenitor cells to regenerate tissues and counter damage from congenital defects either by implantation or facilitating the body's on stem cell populations, how does the authors' message that regeneration is a means to ameliorate damage from congenital defects differ from the established concept of the benefits of endogenous stem cell pools as a means of compensating for injures from congenital defects?

2. Several of the genes that the authors examined are in the endochondral skeleton, which is a tissue not found in the finfold. What exactly are the "malformed skeletal elements"? The authors should also provide more descriptive details about the different "degeneration" phenotypes and provide higher magnified images to visualize much better the degeneration phenotypes and the changes in the expression of the TREE reporter.

3. To assess whether the regenerating program is truly activated in the tissues that manifest human congenital defects, the authors should assess TREE activation in the specific tissues in which the congenital defects are known to manifest (endochondral skeleton, heart, etc.) and not just the finfold. Otherwise, the authors should provide good arguments for why assessing only the finfold suffices.

4. If the authors are concluding that the regeneration program is active, then the authors should show the genuine activation of a broader panel of regeneration genes by qRT-PCR/in situ hybridization, for example genes that represent several major developmental gene pathways: Wnt, BMP, Shh as examples. It is possible that only fgf20a is activated (although the expression of fgf20a was never directly assessed, since the GFP is directly controlled by the stress-induced hsp70l promoter), which may not reflect a genuine regeneration response. If only fgf20a is activated, one could argue that it may just reflect a pro-survival mechanism that limits cell death.

5. In the discussion section, paragraph one, the authors suggest that regeneration mechanisms are retained for countering defects during morphogenesis as well as suggest that this is the reason why some vertebrates (such as zebrafish) have conserved their regeneration programs. Considering the several genes chosen cause human congenital defects, couldn't humans have retained some regeneration mechanisms during early life stages (embryo, pre-juvenile, juvenile) as observed in zebrafish? The authors should provide more discussion on this point using examples in which human tissues regenerate to ameliorate disease phenotypes (e.g. skin using its stem cell population, muscle using its satellite cell population, etc.) at least during early stages of the diseases mentioned in this manuscript.

# Author comment: Author response to Decision Letter 2

**DOI:** 10.1371/journal.pbio.3004011.r003

26 May 2026

Attachment Submitted filename: Response to reviewers.docx

# Peer review: Decision Letter 2

**DOI:** 10.1371/journal.pbio.3004011.r004

**Authors:** Ines Alvarez-Garcia

14 Jul 2026

Dear Dr Poss,

Thank you for your patience while we considered your revised manuscript entitled "Buffering of genetic defects in animal development by regeneration programs" for consideration as a Short Report at PLOS Biology. Your revised study has now been evaluated by the PLOS Biology editors, the Academic Editor and one of the original reviewers.

In light of the reviews (attached below), we are pleased to offer you the opportunity to address the remaining points from Reviewer 3 in a revision that we anticipate should not take you very long. We will then assess your revised manuscript and your response to the reviewers' comments with our Academic Editor aiming to avoid further rounds of peer-review. Please also address the data related points stated below my signature.

In addition to these revisions, you may need to complete some formatting changes, which you will receive in a follow up email. A member of our team will be in touch with a set of requests shortly. If you do not receive a separate email within a few days, please assume that checks have been completed, and no additional changes are required.

We expect to receive your revised manuscript within 1 month. Please email us (plosbiology@plos.org) if you have any questions or concerns, or would like to request an extension.

At this stage, your manuscript remains formally under active consideration at our journal; please notify us by email if you do not intend to submit a revision so that we withdraw the manuscript.

**IMPORTANT - SUBMITTING YOUR REVISION**

Your revisions should address the specific points made by each reviewer. Please submit the following files along with your revised manuscript:

1. A 'Response to Reviewers' file - this should detail your responses to the editorial requests, present a point-by-point response to all of the reviewers' comments, and indicate the changes made to the manuscript.

*NOTE: In your point-by-point response to the reviewers, please provide the full context of each review. Do not selectively quote paragraphs or sentences to reply to. The entire set of reviewer comments should be present in full and each specific point should be responded to individually.

You should also cite any additional relevant literature that has been published since the original submission and mention any additional citations in your response.

2. In addition to a clean copy of the manuscript, please also upload a 'track-changes' version of your manuscript that specifies the edits made. This should be uploaded as a "Revised Article with Changes Highlighted " file type.

To submit a revised version of your manuscript, please go to https://www.editorialmanager.com/pbiology/ and log in as an Author. Click the link labelled 'Submissions Needing Revision' where you will find your submission record.

Thank you again for your submission to our journal. We hope that our editorial process has been constructive thus far, and we welcome your feedback at any time. Please don't hesitate to contact us if you have any questions or comments.

Sincerely,

Ines

--

Ines Alvarez-Garcia, PhD

Senior Editor

PLOS Biology

ialvarez-garcia@plos.org

------------------------------------------------------------------------

ETHICS STATEMENT:

Thank you for including the ethics statement. Please also include an approval number.

------------------------------------------------------------------------

DATA POLICY:

You may be aware of the PLOS Data Policy, which requires that all data be made available without restriction: http://journals.plos.org/plosbiology/s/data-availability. For more information, please also see this editorial: http://dx.doi.org/10.1371/journal.pbio.1001797

Note that we do not require all raw data. Rather, we ask that all individual quantitative observations that underlie the data summarized in the figures and results of your paper be made available in one of the following forms:

1) Supplementary files (e.g., excel). Please ensure that all data files are uploaded as 'Supporting Information' and are invariably referred to (in the manuscript, figure legends, and the Description field when uploading your files) using the following format verbatim: S1 Data, S2 Data, etc. Multiple panels of a single or even several figures can be included as multiple sheets in one excel file that is saved using exactly the following convention: S1_Data.xlsx (using an underscore).

2) Deposition in a publicly available repository. Please also provide the accession code or a reviewer link so that we may view your data before publication.

Regardless of the method selected, please ensure that you provide the individual numerical values that underlie the summary data displayed in the following figure panels as they are essential for readers to assess your analysis and to reproduce it:

Fig. 1F; Fig. 2H; Fig. 3D-H; Fig. 4D, E; Fig. S1B; Fig. S2A, B; Fig. S3E, F; Fig. S4B, C and Fig. S5D, E

NOTE: the numerical data provided should include all replicates AND the way in which the plotted mean and errors were derived (it should not present only the mean/average values).

Please also ensure that figure legends in your manuscript include information on WHERE THE UNDERLYING DATA CAN BE FOUND, and ensure your supplemental data file/s has a legend.

Please ensure that your Data Statement in the submission system accurately describes where your data can be found.

------------------------------------------------------------------------

CODE POLICY

Per journal policy, if you have generated any custom code during the course of this investigation, please make it available without restrictions. Please ensure that the code is sufficiently well documented and reusable, and that your Data Statement in the Editorial Manager submission system accurately describes where your code can be found. More information on our Code Policy, what and how to share can be found here: https://journals.plos.org/plosbiology/s/code-availability

Please note that we cannot accept sole deposition of code in GitHub, as this could be changed after publication. However, you can archive this version of your publicly available GitHub code to Zenodo. Once you do this, it will generate a DOI number, which you will need to provide in the Data Accessibility Statement (you are welcome to also provide the GitHub access information). See the process for doing this here: https://docs.github.com/en/repositories/archiving-a-github-repository/referencing-and-citing-content

----------------------------------------------------------------

Reviewers' comments

Rev. 3:

The proposed hypothesis that regeneration responses are activated by genetic perturbations that generate congenital defects and may explain differences in expressivity and penetrance is important. The data using the zebrafish larva is convincing and supports the authors conclusions that a meaning part of the regeneration program is activated in response to targeting genes associated with human congenital defects. However, the question remains does this occur in clinical models that reproduce the congenital defects, which is an important point of this manuscript. The authors provide supporting arguments from the literature that humans (clinical aspect of congenital defects) do have organs and organ systems that have regeneration capacity, but the brief descriptions of the supporting information do not provide direct evidence that a congenital defect is ameliorated/compensated for by an activated "broader regenerative response" and not just continuous homeostatic maintenance (e.g. the continual generation of skin from the basal stem cells).

In this regard, my original point 3 applies: "To assess whether the regenerating program is truly activated in the tissues that manifest human congenital defects, the authors should assess TREE activation in the specific tissues in which the congenital defects are known to manifest (endochondral skeleton, heart, etc.) and not just the finfold. Otherwise, the authors should provide good arguments for why assessing only the finfold suffices." The authors addressed this point with an explanation for how their hypothesis came to them and with a description about the ease in characterizing the larval regeneration response of the finfold. However, they did not provide an assessment (their own experiment or data from the literature) on whether there is a regeneration response to the known clinically related (e.g. mouse model) contexts of the Fraser syndrome congenital defect or the other human-disease genes they used to show Fgf20a-TREE activation in figure 2. If there isn't citable experimental data in the literature that directly applies to the authors hypothesis, it is likely that there is deep-sequencing data from one or more of the clinical models of the congenital-defect genes that the authors tested in larval fish. How much of regeneration response of the zebrafish larva is similar to the expression data from the clinical model? One possible comparison would be the tbx5 defects: transcriptomic analyses were performed in fish and mouse embryos, so such a comparison with existing data should be possible. The more similarities in the transcriptional responses between the fish and the clinical model, the more the authors can claim that mutation-induced tissue instabilities of congenital defects lead to (are compensated by) activated regeneration responses. As shown in figure 3, If comparable data in the literature can be found, the authors have the ability to make these comparisons, so it should only require a minor revision.

In regards to my original second point (Point 2): The applicable clinical manifestation of Fra1/FRA1 gene defect is skin blistering. The resolution of the images of the skin phenotype are still not high enough to observed this. Do the authors observe blistering in the zebrafish larva skin as observed in clinical models? How much of the clinical phenotype of the skin are manifested in the larva? How do they think the regeneration response affects this?

In Figure 3H, it seems as though there are relatively few categories for such a large number of gene changes. It is also unclear why there is so little overlap among these categories. The authors should provide a supplemental list of the genes that have changed and should organize this list to the categories they described.

# Author comment: Author response to Decision Letter 3

**DOI:** 10.1371/journal.pbio.3004011.r005

14 Aug 2026

Attachment Submitted filename: Response to reviewers Revision 2.docx

# Editor report: Decision Letter 3

**DOI:** 10.1371/journal.pbio.3004011.r006

**Authors:** Ines Alvarez-Garcia

8 Sep 2026

Dear Dr Poss,

Thank you for the submission of your revised Short Report entitled "Regeneration programs buffer genetic defects in animal development" for publication in PLOS Biology. On behalf of my colleagues and the Academic Editor, Philipp Niethammer, I am delighted to let you know that we can in principle accept your manuscript for publication, provided you address any remaining formatting and reporting issues. These will be detailed in an email you should receive within 2-3 business days from our colleagues in the journal operations team; no action is required from you until then. Please note that we will not be able to formally accept your manuscript and schedule it for publication until you have completed any requested changes.

Please take a minute to log into Editorial Manager at http://www.editorialmanager.com/pbiology/, click the "Update My Information" link at the top of the page, and update your user information to ensure an efficient production process.

PRESS

We frequently collaborate with press offices. If your institution or institutions have a press office, please notify them about your upcoming paper at this point, to enable them to help maximise its impact. If the press office is planning to promote your findings, we would be grateful if they could coordinate with biologypress@plos.org. If you have previously opted in to the early version process, we ask that you notify us immediately of any press plans so that we may opt out on your behalf.

We also ask that you take this opportunity to read our Embargo Policy regarding the discussion, promotion and media coverage of work that is yet to be published by PLOS. As your manuscript is not yet published, it is bound by the conditions of our Embargo Policy. Please be aware that this policy is in place both to ensure that any press coverage of your article is fully substantiated and to provide a direct link between such coverage and the published work. For full details of our Embargo Policy, please visit http://www.plos.org/about/media-inquiries/embargo-policy/.

Many congratulations and thanks again for choosing PLOS Biology for publication and supporting Open Access publishing. We look forward to publishing your study.

Sincerely,

Ines

--

Ines Alvarez-Garcia, PhD

Senior Editor

PLOS Biology

ialvarez-garcia@plos.org
